# Does comprehensive education reduce health inequalities?

**DOI:** 10.1016/j.ssmph.2021.100834

**Published:** 2021-06-06

**Authors:** Frank Popham, Cristina Iannelli

**Affiliations:** Moray House School of Education and Sport, The University of Edinburgh, Holyrood Campus, Edinburgh, EH8 8AQ, UK

**Keywords:** Health inequality, Education policy, Tracking, Comprehensive schooling

## Abstract

This article analyses the impact of comprehensive education on health inequalities. Given that education is an important social determinant of health, it is hypothesised that a more equitable comprehensive system could reduce health inequalities in adulthood. To test this hypothesis, we exploited the change from a largely selective to a largely comprehensive system that occurred in the UK from the mid-1960s onwards and compare inequalities in health outcomes of two birth cohorts (1958 and 1970) who attended either system. We studied physical and mental health, health behaviours and life satisfaction in middle age as outcomes and absolute and relative inequalities by social class (of origin and destination) and education. Inverse probability weighting was used to control confounding by socio-economic and education background, and ability test score taken prior to secondary school entry. We did not find consistent evidence that health inequalities were smaller under the comprehensive compared to the selective system and the results were robust under different model specifications. Our study adds to the sparse but growing literature that assesses the impact of social policy on health inequalities.

## Highlights

• Comprehensive schooling aims to reduce educational opportunity inequalities.
• Reducing educational opportunity inequalities may reduce health inequalities.
• Natural experiment study of change from tracked schooling in UK.
• Little evidence of impact on health inequalities in middle age.
• Extensive sensitivity analysis conducted.

## 1 Introduction

Although life expectancy has increased over recent decades, in many western countries health inequalities between socio-economic groups persist (Mackenbach., 2012). Existing research on these inequalities analyses their social determinants often in a rather descriptive way. It has been argued that research needs to move beyond description to study whether social policies that affect the social determinants can also impact health inequalities (Beckfield & Krieger, 2009). Education is an important social determinant of health and it is seen as a possible policy area through which health inequalities could be reduced (Jones et al., 2014). This paper contributes to a sparse but growing literature on the role of education policies for health inequalities by examining whether the introduction of comprehensive secondary education, using the example of Britain, has led to any change in health inequalities measured by a variety of both objective and subjective indicators. Countries vary in the way in which they organize their secondary school system and allocate students within it. Some countries separate students between tracks, academic and vocational orientated schools, while others teach all students of all abilities in the same comprehensive schools, although they may stream by ability within schools. While the equality impact of the different systems on educational and labour market outcomes has been extensively studied (Burger, 2016; Burgess et al., 2020) to date only a few studies have explored the health equality impacts of different tracking systems (Rathmann et al., 2016; Meghir et al., 2013; Ravesteijn et al., 2017; Delaruelle, Van Houtte, & Bracke, 2019; Delaruelle, van de Werfhorst, & Bracke, 2019). We might expect that more equitable education systems help to reduce health inequalities by reducing inequalities in resources that are important for health (section 2.3 provides an explanation of potential mechanisms). Thus, in our paper we ask the following research question: did the introduction of a comprehensive education system in Britain reduce health inequalities?

Before addressing this question empirically, we discuss the literature on the importance of tracking for inequalities and the reasons why a change from a selective to a comprehensive school system might impact social inequalities and health inequalities in particular.

## 2 Theoretical background and existing empirical evidence

### 2.1 The relation between education institutions and social inequalities

Theories of social reproduction have long argued that the organization, selection procedures and content of the school system are very important factors in the explanation of the unequal distribution of education and labour market outcomes. In particular, the school factor which has most strongly been accused of reproducing social inequalities is track allocation; that is the allocation of pupils into different schools, or programmes of study within schools, based on student ability or performance. Tracks have different status and curriculum content: usually academic schools/programmes have higher status and lead to more advanced studies and vocational schools/programmes have lower status, a narrower curriculum and limited or no opportunity for further studies. A large number of sociological and educational studies have been devoted to analysing the role of tracking and its effect in widening, reducing or simply maintaining existing inequalities among students from different social classes (Gamoran, 1996; Gamoran & Mare, 1989; Kerckhoff, 1986; Oakes, 1985). They generally found that low tracks restrain the educational and socio-economic attainments of disadvantaged pupils with long-term negative consequences on individuals’ lives.

After the 1944 Education Act that provided for universal free secondary education, Britain had a largely tracked secondary school system - the tripartite system-with its distinction between grammar, technical and secondary modern school. Pupils were separated into different schools according to their ability which was determined by the results of the 11-plus exam at the end of primary school: those who achieved the best exam results gained access to grammar school, which taught a highly academic curriculum; the others attended secondary modern and, a small minority, technical schools which offered a vocationally-oriented curriculum. There were also a number of independent, fee-paying schools which selected their pupils on the basis of entry tests and financial criteria.

Critics of the tripartite system argued it reproduced societal inequalities since selection based on the 11-plus discriminated against children from disadvantage socio-economic backgrounds and resulted in their over-representation in the secondary modern schools. Indeed, the move to a comprehensive system from the mid-1960s onwards was partly advocated by the aspiration to provide more equal educational opportunities. Among the purposes of the comprehensive re-organization of the education system were the elimination of pupils' allocation to schools through selection by ability, a reduction of between-school variation in children's intake (favouring a more diverse social mix in schools), an increase in access to educational certifications, and a reduction of social inequalities in attainment (Kerckhoff, 1996; McPherson & Willms, 1987).

The implementation of the comprehensive reform was uneven across Great Britain. This was because the government circulars 10/65 (England and Wales) and 600 (Scotland) invited Local Education Authorities (LEAs) to submit their plans for the comprehensive reorganisation of the education system. While LEAs in Scotland and Wales moved relatively quickly to a full comprehensive system, there was more resistance in England where several LEAs decided to continue maintaining their selective system and in some cases grammar schools coexisted (and continue to coexist to the present days) alongside comprehensive schools (Pring, 2015). In comprehensive secondary schools in Britain, all pupils aged 11 to 16 are taught a general curriculum which includes both academic and vocational subjects.

### 2.2 The empirical evidence on comprehensive education and inequalities

The issue of whether comprehensive education systems are more equitable than selective education systems has been prominent in the education and social mobility literature. Some scholars have tried to answer this question by comparing countries with different degrees of differentiation of their education systems (Burger, 2016; Dupriez & Dumay, 2006; Gorard & Smith, 2004; Van de Werfhorst & Mijs, 2010), while others have concentrated on within-country comparisons to analyse changes in national education systems over time (Boliver & Swift, 2011; Heath & Clifford, 1990; Iannelli & Paterson, 2007; Kerckhoff, 1996). In general, results from the international comparisons suggest that comprehensive systems (as those in the Scandinavian countries), where there is no selection by ability and all pupils attend the same type of schools, are more egalitarian than differentiated school systems (such as those in Germany, Austria and Switzerland), which are characterised by sorting pupils according to their ability in different school types or tracks.

Interestingly, research based on within-country comparisons has mostly concluded that the comprehensive reorganisation of national education systems had little impact on reducing social inequalities in attainment and in promoting social mobility. Atkinson et al. (*2006*) found that, overall, pupils in selective Local Education Authorities (LEAs) in England did not achieve better educational outcomes than similar pupils in non-selective LEAs. This is because while children educated in grammar schools outperformed similar children educated in comprehensive schools, non-grammar-educated children in the selective system, which mostly gathered children from less advantaged social backgrounds, underperformed them.

Examining social mobility chances in Britain, Boliver and Swift (2011) found that the selective system was no better than the comprehensive system in improving the chances of children from disadvantaged social origins of being upwardly mobile. In Scotland, where the comprehensive reorganisation of the education system was more complete than in England, Iannelli and Paterson (2007) compared four cohorts born between 1937 and 1975 and found no evidence that the introduction of comprehensive education in Scotland was associated with improved social mobility. Conversely, Burgess et al. (2020) analysed earnings inequality between people who attended the selective and the comprehensive system and found that individuals who grew up in areas operating a selective school system had a more unequal earnings distribution.

Much less explored is the issue of whether comprehensive education may lead to benefits in life domains other than education and labour market outcomes. Another social impact that comprehensive schooling has been hypothesised to have is to improve social cohesion. To test this, studies have analysed the relationship between having attended comprehensive schools and individuals’ civic values. As for the above-mentioned studies, cross-country comparative and single-country research reach different conclusions. While comparative research observed a positive relationship, with people who attended comprehensive systems showing more civic-minded views (van de Werfhorst, 2007), single-country analysis of longitudinal data found that those who attended selective schools tended to be more liberal, tolerant and engaged in politics than people who attended comprehensive schools (Paterson, 2012).

### 2.3 Reasons why comprehensive education might reduce health inequalities

A number of prominent theories of health inequalities predict that more equal distribution of the socio-economic determinants of health should lead to reduced health inequalities (Phelan et al., 2010; Towsend et al., 1990; Wilkinson & Pickett, 2008). Fundamental Cause theory offers an overarching theory for the persistence of health inequalities over time, place and generation. It argues that “individuals and groups deploy resources to avoid risks and adopt protective strategies. Key resources such as knowledge, money, power, prestige, and beneficial social connections can be used no matter what the risk and protective factors are in a given circumstance.” (Phelan et al., 2010, p. 29). It argues for studying the role of stratification systems, such as the schooling system in the perpetuation of these resource inequalities. Below we give three theoretical explanations which can explain the way a comprehensive school system might reduce resource inequalities leading to reduced health inequalities. The interested reader is also directed to Delaruelle, van de Werfhorst, and Bracke (2019).

1) Knowledge resources

One way that the comprehensive system might affect health is through reduced knowledge disparities given a universal and more academically focused curricula in the comprehensive school system. For example, Mirowsky and Ross argue that education enhances the individual's ability to effectively navigate risk and be resilient, and thus improve health, what they term “learned effectiveness” (Mirowsky & Ross, 2005). Relatedly, the multidimensional concept of health literacy is routinely found to be associated with more education. Education may then improve seeking, comprehending and actioning health knowledge as well increasing the ability and power to navigate the health system effectively (Beauchamp et al., 2015; Greenhalgh, 2015; van der Heide et al., 2013). Causal studies on the relationship between education and high literacy are mixed. For example, a UK natural experiment study using changes in compulsory school leaving age did not find a causal relationship between increased leaving age and health knowledge while a similarly designed Australian study found that some, but not all, knowledge informed health behaviours were improved by increasing the school leaving age (Johnston et al., 2015; Li & Powdthavee, 2015).

2) Socio-economic resources

Access to money, power and prestige are linked to occupational class and status commonly derived through obtaining educational qualifications. Thus, a comprehensive school system, by improving the education and labour market outcomes of the most disadvantaged, may reduce health inequalities by narrowing the socio-economic resource gap. Indeed, social mobility research in the UK has found that in the second half of the 20th century, absolute rates of mobility were high, meaning that a large number of working-class children were upward mobile and entered professional and managerial occupations. This was mostly due to the expansion of professional occupations and of education participation which occurred in this period (Goldthorpe & Mills, 2004; Iannelli & Paterson, 2006). Heller and colleagues found evidence that the changing occupational structure in the UK to more high-status jobs had benefited population health in the 1990s compared to the 1970s (Heller et al., 2002). On the other hand, and as already highlighted, research generally finds that relative social mobility did not greatly change after the switch to a largely comprehensive system. Evidence on the impact of relative social mobility on health inequalities is mixed with evidence of both a role in reducing and increasing health inequalities (Bartley & Plewis, 2007; Boyle et al., 2009).

3) Connectedness resources

As highlighted, improved social cohesion is theorised as being a possible outcome of moving to a comprehensive system as pupils from different social backgrounds are more integrated. In health Wilkinson and Pickett's income inequality theory hypothesises “that more equal societies were healthier because they were more cohesive and enjoyed better social relations.” They argue that the main biological mechanism by which social cohesion improves health is through the lowering of stress by the diminishing of prestige and status hierarchies, as chronic stress can impair health. For rich countries, they argue that it is the distribution rather than the average level of socio-economic resources that is important for health (Pickett & Wilkinson, 2015). So, if the comprehensive system does improve social cohesion, as theorised, this could be another route through which it reduces health inequalities.

### 2.4 Previous studies on tracking and health inequalities

Two recent cross-national studies analysed differences in self-rated health by school level tracking. The first found worse self-rated health amongst those graduating with, at a similar level, vocational compared to academic qualifications and these differences were more pronounced in countries with more tracked school systems (Delaruelle, Van Houtte, & Bracke, 2019). The second found that health inequalities between young people with different educational achievement were slightly larger under comprehensive rather than more selective systems as more advantaged young people's health improved but that of the more disadvantaged worsened slightly under the comprehensive system (Delaruelle, van de Werfhorst, & Bracke, 2019). In relation to young people's smoking a cross national study found that relative health inequalities were not greater in countries with more selective education systems (Rathmannn et al., 2016).

A Finnish study found that later tracking improved survival at age 50 for those from low income backgrounds but reduced survival for those from high income backgrounds, thus reducing inequalities (Ravesteijn et al., 2017). A Swedish study found some small effects of the introduction of comprehensive school including reducing mortality and improving health amongst those from disadvantaged backgrounds but overall impacts on population health were not seen (Meghir et al., 2013).

### 2.5 The current study

Our paper aims to provide new evidence on whether a comprehensive education system may reduce health inequalities. One limitation of cross-country comparative studies is the difficulty in establishing causal relations between institutional features of national education systems and inequalities of outcomes. This is because national institutions and policies are embedded in and interact with broader socio-cultural contexts which greatly differ among countries. Hence, we follow other researchers (Boliver & Swift, 2011; Paterson, 2012) and focus on institutional changes within one country, Britain. Of course, it is worth noting that also the results of single country studies may be confounded if other socio-economic changes co-occur as the school system transitions (Van der Werfhorst, 2019). Previous within country studies involve comparison across cohorts. We add to the literature by exploiting the fact that the old selective and the new comprehensive systems co-existed (and continue to co-exist in parts of England) and this allowed us to examine the health outcomes of young people in the same cohort, thus keeping constant other period and contextual effects.

## 3 Methods

A protocol for the study was registered before analysis of the outcome data was undertaken (https://osf.io/vt46r).

### 3.1 Design

To obtain an average population effect of school system on health and health inequalities we contrasted the outcomes between the Comprehensive and Selective systems using observational data. Given the lack of randomisation in school system allocation, we controlled for individuals pre-secondary school characteristics associated with both school type and later health including sex, ethnicity, region of residence, cognitive ability, and childhood socio-economic circumstances. We did this by modelling school system probability as a function of the confounders. From these probabilities we calculated inverse probability weights to try to balance the confounders across the two school systems (Hernán & Robins, 2020). In our main analysis we used Superlearner to select the best balancing weights from three methods of fitting the models of school system (logistic regression, generalized additive model and generalized linear model via penalized maximum likelihood) (Pirracchio et al., 2015). In sensitivity analysis we also separately modelled school system as a function of confounders using a generalized boosted model and entropy balancing.

### 3.2 Data

We used data from two UK longitudinal birth cohorts that have surveyed one week of births from 1958 to 1970 regularly throughout their lives. The 1958 cohort is known as the National Child Development Study and the 1970 as the British Birth Cohort Study; for ease we refer to them as the 1958 and 1970 cohorts respectively.

We used data from both cohorts because, while the first cohort went to school during the transition from a selective to a comprehensive system, the second cohort experienced a more ‘mature’ comprehensive system. Indeed, in Scotland and Wales, by 1980 almost all pupils in state-funded schools were attending schools that were comprehensive while in England some areas retained a selective system of secondary school (Croxford, 2004). This allowed us to test the robustness of our results and to reach more confident conclusions.

Both cohorts are described in detail elsewhere (Power & Elliott, 2006; Elliott & Shepherd, 2006). The cohorts are regularly surveyed as were their parents and schools in childhood. Both cohorts added a small number of immigrants to the UK, who were born in the same week outside the UK, by tracing through their schools (Power & Elliott, 2006; Elliott & Shepherd, 2006) but we exclude this group as their school age data is often missing. Additionally, we exclude the small number of respondents born in Northern Ireland as they were not followed into adulthood. So our sample in both cohorts was those born, alive and living in Great Britain at the survey wave just before secondary school (aged 10 in 1970 and aged 11 in 1958).

#### 3.2.1 Exposure

Our exposure is the selective system (grammar, private and secondary modern schools) versus the comprehensive system. In the 1958 and 1970s study school type was reported at age 16 (end of compulsory secondary schooling) rather than age 11 (start of secondary schooling). Especially in the 1958 cohort, schools may have converted to being a comprehensive after the cohort member started secondary school. As the 1958 cohort reports the year the school changed to a comprehensive, we conducted sensitivity analysis using school type at age 11 in this cohort. In the 1970 cohort there was a teachers’ strike during the age 16 wave of data collection affecting the level of response, so the cohort was asked about secondary school type at age 42 as well to supplement the age 16 data.

#### 3.2.2 Outcome measures

##### 3.2.2.1 Health and wellbeing

Our six health and wellbeing outcomes are body mass index (BMI), smoking behaviour, self-rated health, wellbeing measured using the Warwick-Edinburgh Mental Wellbeing Scale (WEMWBS) and life satisfaction (past and future). They were all collected when cohort members were aged 42 with the exception of WEMWBS which was collected at age 50 for the 1958 cohort.

We chose two health behaviours as it has been hypothesised that Fundamental Cause theory will apply more strongly for preventable causes of disease through knowledge resources (Mackenbach et al., 2017). Both the health impact of excess body fat and smoking have been known for some time. Inequalities in these health behaviours have been ascribed to differences in socio-economic and connectedness resources as well (Hiscock et al., 2012; Pickett & Wilkinson, 2015).

Self-rated general health is an established measure of general health that predicts mortality, although there remains debate about what exactly it captures and how this varies by context (Jylhä, 2009). The WEMWBS scale captures the following aspects of mental wellbeing in a single construct: “hedonic and eudaimonic aspects, positive affect, satisfying interpersonal relationships and positive functioning” (Tennant et al., 2007). Life satisfaction is a widely used evaluative measure of well-being and is used by government as one measure of national well-being in England and Wales (Tinkler & Hicks, 2011). BMI at baseline will be used as a negative control in sensitivity analysis (Lipsitch et al., 2010). We used outcome data from cross-cohort harmonised datasets designed for comparative analysis of our cohorts when this was available (Wood et al., 2019; Hardy et al., 2019).

##### 3.2.2.2 Social class

Parental social class was measured when cohort members were aged 10 or 11. We refer to this as origin social class. Cohort members' adult social class (destination social class) was measured at age 42. As our measure of social class we used the UK's National Statistics Socio-economic classification of occupations (NS-SEC) (Office for National Statistics, 2016). We used the NS-SEC codes derived for comparative analysis by a previous cross-cohort project (Morris, 2012). As this did not have age 42 NS-SEC coding for the 1970 project we used NS-SEC coding provided by the 1970 study team instead. Our measure of inequality requires an ordered measure of social class. We used the three-category ordered version of NS-SEC (Office for National Statistics, 2016), and further split the most advantaged category into two. Thus, we use the following four category ordered classes: higher managerial and professional occupations, lower managerial and professional occupations, intermediate occupations, routine and manual occupations (Duta et al., 2021).

##### 3.2.2.3 Education

Using education files created for comparative analysis across our cohorts we included the age of leaving full time education and highest qualification by age 38 as outcomes and potential mediators of any relationship between school system and health inequality. Those with highest qualifications of 1–4 O-level passes, NVQ 2 or below (including no qualifications) were coded as “below high lower secondary.” “High lower secondary” included those with the highest qualifications of 5+ O-level passes or 1 A-level pass or NVQ 3. “Higher secondary or lower tertiary” covered those with tertiary level sub-degree qualifications, NVQ 4 or 2+ A-level passes. Finally those with a degree or NVQ 5 and above were coded “higher tertiary” (Bourne et al., 2018).

#### 3.2.3 Confounders

We chose factors at baseline that could influence the school system attended and later health. These were region of residence, cognitive ability, sex, ethnicity, origin social class, parental education and interest in their child's education (reported by teachers), and whether the pupil attended a state funded primary school. Tables 1a, Table 1ba and 1b lists the categories of these confounders. Region of residence recorded at baseline used standard region classifications in operation at the time and varied slightly between the cohorts. Ethnicity was captured at age 42 in the 1958 cohort as it was not well covered in childhood surveys, and at age 5 in the 1970 cohort. Low prevalence of some ethnicities in both cohorts meant quite broad categories were used. In the selective system the type of school one attended (academic or vocational focused) was based on an entrance exam taken at the end of primary schooling. While not the test themselves, the 1958 and 1970 cohorts took similar cognitive ability tests at age 11 and 10 respectively that correlate highly with the school admission tests (Parsons, 2014; Shepherd, 2012). The 1958 cohort test was the General Ability teacher administered test that consisted of 40 verbal and 40 non-verbal questions (Shepherd, 2012). Pupils' verbal and non-verbal abilities were tested in the 1970 cohort through the use of four sections of the British Ability Scores test. Again the test was teacher administered (Parsons, 2014). For both cohorts we standardised the scales to have a mean of 100 and standard deviation of 15. Parental education was based on the highest of either parents' age at leaving school. From a cross-cohort dataset derived for comparative analysis we included primary schools' rating of parents' interest in their child's education at age 7 for the 1958 cohort and age 10 for the 1970 cohort (Wood et al., 2019). Finally, parents were asked when their child was aged 11 in the 1958 cohort and aged 10 in the 1970 cohort what age they thought their child would leave school and if they hoped they would stay in education post school.

**Tables 1a:** (1958) and b (1970): Confounder balance before and after weighting (%).

			Before		After	
		All	Comp	Select	Comp	Select
**A) 1958**	**N**	**15,715**	**9,428**	**6,287**	**15,703**	**15,588**
Sex	Female	48.9	48.5	49.4	48.9	48.6
	Male	51.1	51.5	50.6	51.1	51.4
Ethnicity	Asian	0.2	0.2	0.1	0.2	0.2
	Black	0.5	0.6	0.3	0.5	0.5
	Other	0.6	0.7	0.5	0.6	0.6
	White	98.7	98.5	99.1	98.7	98.7
Cognitive ability	(mean)	100.2	98.0	103.3	100.1	100.2
Region of residence	E & W.Riding	8.4	10.9	4.6	8.4	8.3
	East	8.6	8.4	8.9	8.6	8.7
	Midlands	9.3	8.4	10.7	9.3	9.3
	North	6.9	6.8	7.0	7.0	7.1
	North Midlands	7.8	7.0	8.9	7.9	8.0
	North West	12.9	10.3	16.7	12.8	12.9
	Scotland	10.6	15.9	2.6	10.6	9.5
	South	6.3	4.9	8.4	6.3	6.4
	South East	17.6	14.2	22.8	17.5	17.7
	South West	6.1	5.6	6.9	6.1	6.2
	Wales	5.5	7.5	2.5	5.5	5.8
Age parent left education	15 or below	73.2	78.8	64.8	73.5	72.9
	16 to 18	21.3	17.9	26.5	21.1	21.3
	19 plus	5.5	3.3	8.7	5.5	5.8
When parents hope will leave school	Don't know yet	19.2	20.4	17.4	19.3	19.5
	Leave at minimum age	5.2	5.7	4.6	5.3	5.4
	Stay on longer	75.5	73.9	78.0	75.4	75.1
Parents hope stays in education post school	Don't know yet	14.2	15.4	12.4	14.3	14.5
	No	3.0	3.1	2.9	3.0	3.2
	Yes	82.8	81.5	84.7	82.7	82.3
Parents interest in school rated by school	Little interest	15.3	17.4	12.2	15.4	15.5
	Some interest	42.8	45.7	38.5	42.9	42.2
	Very interested	41.8	36.9	49.2	41.7	42.3
State primary school	No	5.9	3.8	9.2	5.7	6.0
	Yes	94.1	96.2	90.8	94.3	94.0
Father's NSSEC	Higher managerial	15.5	11.6	21.4	15.4	15.9
	Intermediate	21.5	21.3	21.9	21.6	21.4
	Lower managerial	4.4	3.5	5.7	4.3	4.4
	Routine	58.6	63.7	51.0	58.8	58.3

**Table 1b:** (1970): Confounder balance before and after weighting (%).

			Before		After	
		All	Comp	Select	Comp	Select
B) 1970	N	15,699	13,368	2,332	15,693	15,499
Sex	Female	48.5	48.4	48.8	48.5	48.9
	Male	51.5	51.6	51.2	51.5	51.1
Ethnicity	European UK/Other	96.5	96.4	97.1	96.4	96.4
	Indian/Pakistani/other Asian	1.9	2.0	1.2	1.9	1.8
	Other	0.3	0.3	0.1	0.3	0.3
	West Indian/African	1.4	1.3	1.5	1.4	1.5
Cognitive ability	(mean)	100.0	99.0	105.5	100.0	99.9
Region of residence	East Anglia	3.4	3.6	2.3	3.4	3.1
	East Midlands	7.1	7.3	6.1	7.2	7.3
	North	6.1	6.7	2.4	6.0	4.8
	North West	12.6	12.9	10.6	12.5	12.2
	Scotland	9.6	9.5	10.6	9.8	11.1
	South East	28.6	26.2	42.6	28.6	29.7
	South West	7.5	7.2	9.8	7.5	7.7
	Wales	5.5	5.9	2.9	5.5	5.4
	West Midlands	10.2	10.8	7.0	10.2	9.9
	Yorks and Humberside	9.3	9.9	5.7	9.3	8.9
Age parent left education	15 or below	52.2	54.7	37.7	52.3	51.7
	16 to 18	33.5	33.1	36.1	33.5	33.9
	19plus	14.3	12.2	26.2	14.2	14.3
When parents hope will leave school	16 years old	43.2	45.7	28.6	43.3	42.6
	17 years old	15.6	15.9	14.4	15.7	16.6
	18 years old	41.2	38.4	57.0	41.0	40.8
Parents hope stays in education post school	Cannot say	55.6	57.8	42.9	55.7	55.3
	No	4.7	5.0	2.9	4.7	4.8
	Yes	39.7	37.2	54.2	39.6	39.9
Parents interest in school rated by school	Little interest	9.5	10.0	6.5	9.5	9.7
	Some interest	35.8	37.3	27.2	35.9	36.0
	Very interested	54.7	52.7	66.3	54.6	54.3
State primary school	No	3.7	1.5	16.4	3.6	3.7
	Yes	96.3	98.5	83.6	96.4	96.3
Father's NSSEC	Higher managerial	19.4	17.2	32.0	19.3	19.7
	Intermediate	21.8	22.0	20.8	21.8	22.4
	Lower managerial	5.7	5.4	7.3	5.7	5.4
	Routine	53.1	55.4	39.9	53.2	52.5

### 3.3 Analysis

#### 3.3.1 Inequality

Our main outcome measure is social class inequalities in the health outcomes. As our measure of inequality, we used the concentration index (Erreygers & Van Ourti, 2011). This measures deviation from the line of equality where the ranked share of socio-economic groups in the population have an equal share of the health outcome. It is related to the slope and relative index of inequality also commonly used in studies of health inequalities (Kjellsson et al., 2015). Three measures will be studied: absolute inequality, and “attainment” and “shortfall” relative inequality. We use two measures of relative inequality as the results can vary depending on whether the outcome is coded as positive (attainment) or negative (shortfall) health (Søgaard & Lindholt, 2019).

To calculate the concentration index we used regression models as outlined below. The independent variable in the regression was the socio-economic measure we were using to assess inequality. The socio-economic measure is ranked from the most disadvantaged to the most advantaged with each group coded at the mid-point of their ranked cumulative proportion (O'Donnell et al., 2016). For example, if our socio-economic measure was social class with five ranked class groups of equal size, the groups would be coded from disadvantaged to advantaged as 0.1, 0.3, 0.5, 0.7, 0.9.

The dependent variable in the regression was the health outcome (or other outcome of interest). First, each of our outcomes was recoded as both positive (attainment), from worse to best, and negative (shortfall), best to worse. Then each outcome was scaled to 0 to 1 to make the concentration index independent (scale invariant) of the different measurement scales of the outcomes (Erreygers & Van Ourti, 2011). For the relative concentration indices, the outcome was then multiplied by twice the variance of the independent variable (i.e. the recoded socio-economic variable described in the last paragraph). It was then divided by the mean of the outcome (O'Donnell et al., 2016). For the absolute measure, the relative dependent variable was multiplied by 4 times the mean of the outcome (O'Donnell et al., 2016).

To calculate the concentration indices, the dependent and independent variables described above were entered as continuous variables in a linear regression. To assess whether the index varies by school system, we interacted the independent variable with a dummy variable for school system (O'Donnell et al., 2016).

All calculations included the inverse probability weights to control for confounding.

#### 3.3.2 Missing data

As with any cohort study, missing data due to attrition (non-response to survey) and item non-response are common in both cohorts. We used a two stage MI/IPW approach to address it (Seaman et al., 2012). Details in Supplement 1.

#### 3.3.3 Software

For the analysis we used R (R Core Team, 2020) with Rstudio (RStudio R Core Team, 2020). We made use of several user written packages for R, these are listed in Supplement 9.

## 4 Results

### 4.1 Baseline

**Fig. 1:**
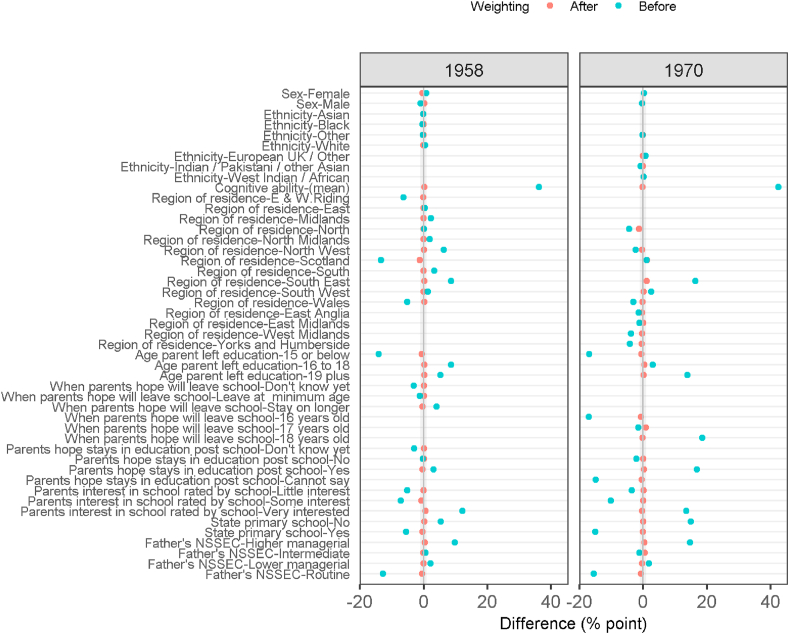
Confounder balance before and after weighting.

Tables 1a, Table 1ba and 1b shows confounder balance before and after adjustment using the inverse probability weights in the 1958 and 1970 cohorts respectively. Just over half of the 1958 cohort were in comprehensive schools at age 16 compared to 83% in the 1970 cohort. Before adjustment on average those attending selective system schools were from more advantaged backgrounds and scored higher on the cognitive ability tests. As illustrated in Fig. 1, good balance was achieved across the confounders in both cohorts after adjustment. In supplement 2 we illustrate good balance after adjustment in the distribution of cognitive ability (the continuous confounder). In addition, Tables 1a, Table 1ba and 1b shows that the adjusted counterfactual populations - imagining everyone went to school under a comprehensive system or a selective system - in both cohorts closely match the overall population in terms of size and variable distribution.

### 4.2 Outcomes

**Fig. 2:**
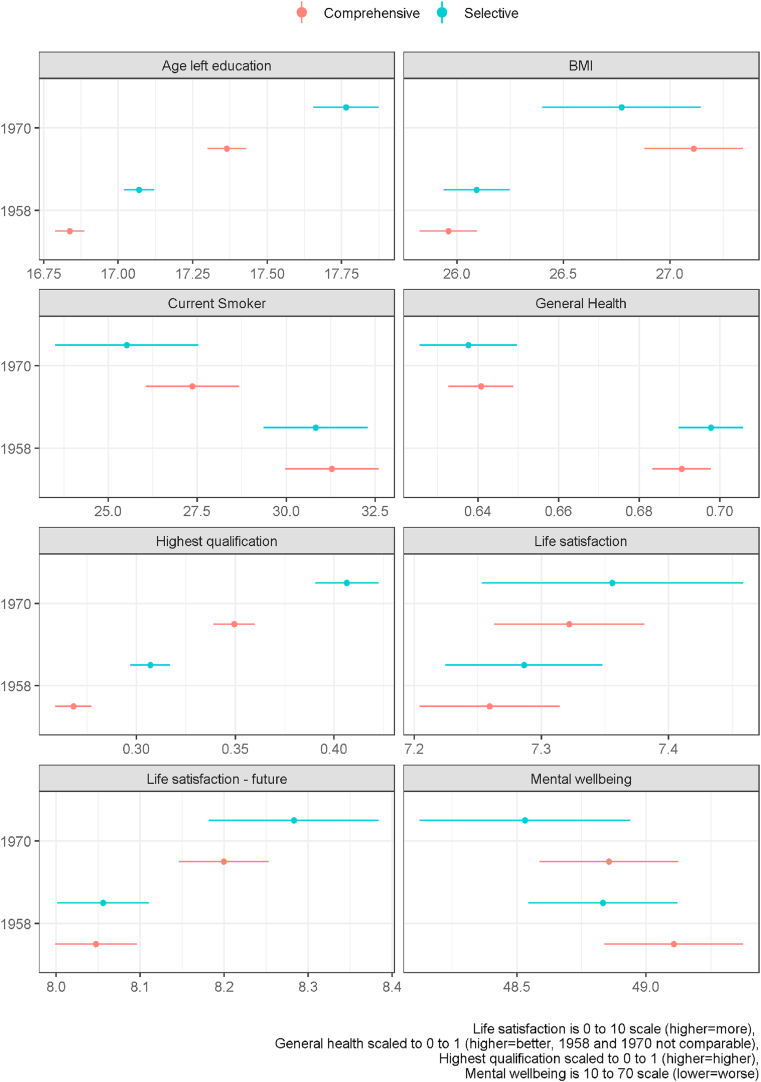
Mean outcomes by school system and cohort.

There are clear differences across the cohorts in the mean of some outcomes - rises in age leaving full time education, increases in qualification levels, increases in BMI and future life satisfaction, and reductions in smoking - as might be expected. For example, there were fewer smokers at age 42 in 1970 from both the comprehensive system (27.3% (95% CI 26%–28.6%)) and the selective system (25.7% (23.7%–27.6%)) compared to 1958 (31.2% (30%–32.5%) and 30.9% (29.4%–32.3%) respectively) as shown in Fig. 2 that illustrates the means of outcomes under each system after confounder adjustment. Although general health seems to have reduced in the 1970 cohort, this is perhaps the least comparable variable due to differences in response scale in the original questions.

Comparing within cohorts, Fig. 2 shows that in general there was not a clear difference between school systems for our health and wellbeing outcomes. For example, while the rate of smoking was slightly lower in both cohorts under the selective system compared to the comprehensive system (−0.4% (−2.4% - 1.6%) in 1958 and -1.7% (−4.1% - 0.7%) in 1970), the confidence intervals for the differences were wide. There were clear differences in the two education outcomes under each system. For example, average age at leaving full-time education was higher in the selective system in both cohorts. In 1958 the average age of leaving full-time education under the comprehensive system was 16.8 (16.8–16.9) while under the selective system it was 17.1 (17–17.1), a difference of 0.23 (0.16–0.30) years, just under 3 months. In 1970 the average age of leaving full-time education under the comprehensive system was 17.4 (17.3–17.4) while under the selective system it was 17.8 (17.7–17.9), a difference of 0.4 (0.27–0.52) years, just under 5 months.

**Fig. 3:**
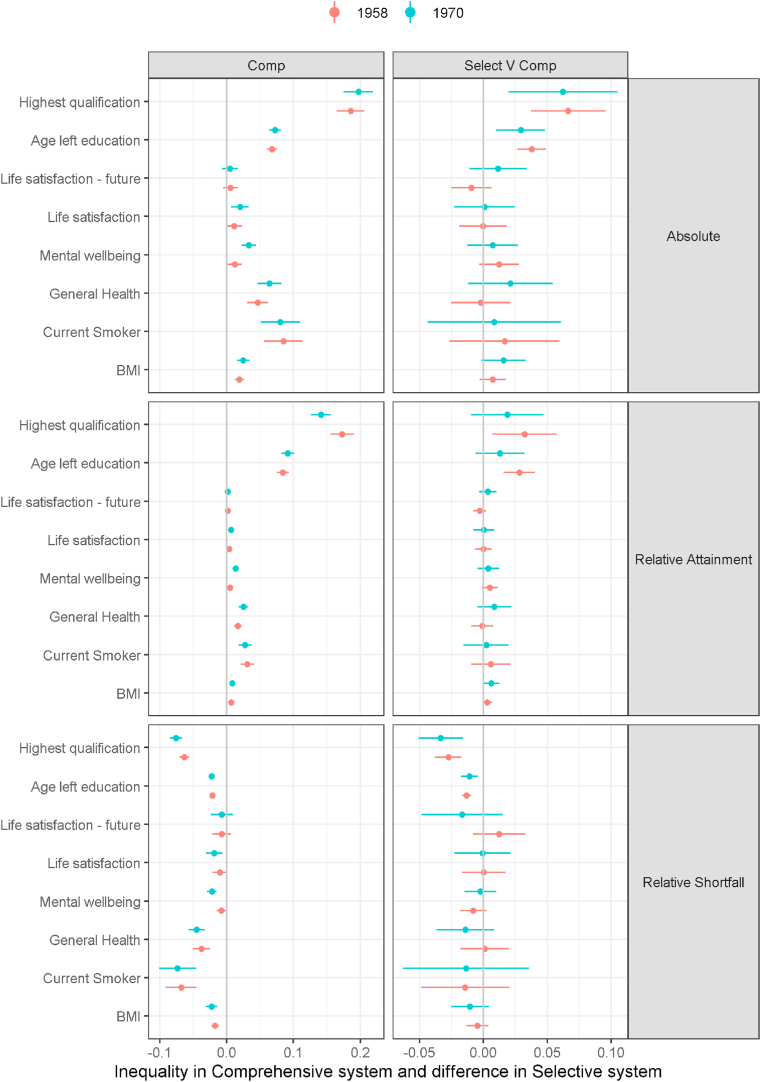
Origin class inequalities in outcomes by school system and cohort.

A similar picture is observed for inequality in outcomes as that for mean outcomes. Fig. 3 shows inequality in the comprehensive system in each outcome by our three measures - i.e. absolute inequality, and shortfall and attainment relative inequality. Additionally, it shows the difference in inequality under the selective system compared to the comprehensive system. It reports inequality by class of origin (results are similar for class of destination, see supplement 3). Overall inequality is not clearly different in the selective system compared to comprehensive system for our health and wellbeing outcomes. For example, the result for mental wellbeing shows inequalities by social origin in the comprehensive school, meaning that people from more disadvantaged social origins tend to have poorer mental wellbeing than people from more advantaged social classes. The results are invariant to whether we consider positive mental wellbeing (relative attainment) or negative mental wellbeing (relative shortfall). Absolute results are presented as attainment only as by definition they are invariant (apart from the sign switching to negative for shortfall). However, this pattern is not greatly different under the selective system. Similar results for general health, BMI, current smoking and present life satisfaction. Inequalities in future life satisfaction are less apparent. For both the education outcomes of age of leaving full-time education and highest education there are clear inequalities - earlier age of leaving and lower qualifications among the more disadvantaged - and the inequalities are generally greater in the selective system. The only exception is the relative attainment measure for both highest qualification and age leaving full-time education in the 1970 cohort where the selective system's difference to the comprehensive system is less clear as the confidence intervals cross zero.

### 4.3 Education inequalities in health outcomes

**Fig. 4:**
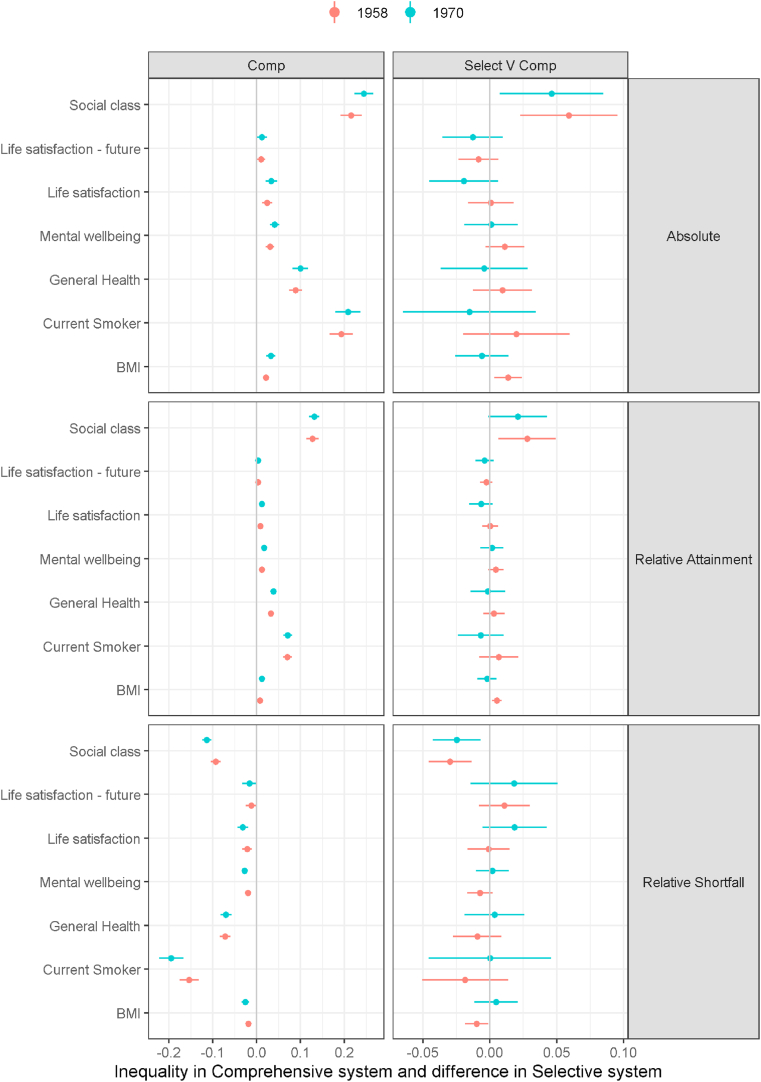
Education inequalities (age left education) in outcomes and social class of destination by school system and cohort.

So far, we have used social class as our measure of stratification. We also explored inequalities with education as the measure of stratification. While we hypothesised that the comprehensive system might influence health inequalities indirectly through occupational social class and associated economic advantage, we also hypothesised that it might have more direct effects on health inequalities, for example through the distribution of health literacy. Thus, if it had a direct effect through education, we might capture this better when using education as a measure of stratification. It is also valuable to assess our outcomes using education as the measure of stratification because inequalities in health are often studied using education rather than social class. Specifically, we report inequalities in health outcomes by the age left full-time education in Fig. 4 which replaces social class as the stratification variable with age left full-time education. Overall, there is little evidence that inequality by education in the health and wellbeing outcomes is different in the selective system compared to the comprehensive, as was the case for social class stratification. The one exception being inequality in BMI that seemed to be greater in the 1958 cohort. Fig. 4 additionally shows education inequality with social class of destination as an outcome. Here there is consistent evidence that social class advantage (attainment inequality) and social class disadvantage (shortfall inequality) are more concentrated by age left education in the selective as opposed to the comprehensive system. Results are similar using highest qualification achieved (see supplement 3).

### 4.4 Exploratory analysis

**Fig. 5:**
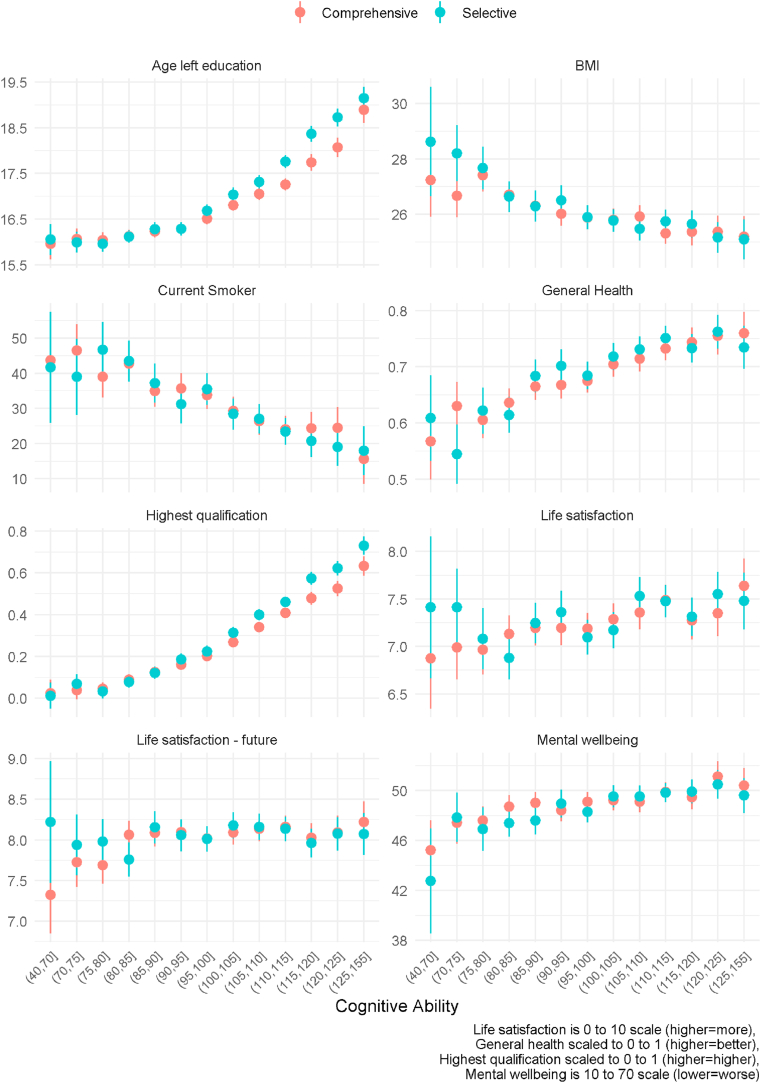
Mean of outcomes by cognitive ability and school system 1958 cohort.

We undertook exploratory analysis to assess why the mean and inequality might be greater in the selective system for education but not health outcomes. The main feature of the selective system is that entry to either a vocational or academic focused secondary school is based on a cognitive ability test taken at the end of primary school. While not the actual test we do have similar cognitive ability tests in our data, so it is informative to compare outcomes by childhood cognitive ability and school system. The results for the 1958 cohort can be seen in Fig. 5. We binned cognitive ability into five-point bins (except at the margins where cases were most sparse) and calculated the mean of the outcome for each bin by school system adjusting for background characteristics using inverse probability weighting. Apart from perhaps the life satisfaction outcomes, the general trend in Fig. 5 is that increasing cognitive ability is associated (no causal claim being made) with better outcomes as previous research suggests. However, only for the education outcomes do we see a difference emerge by system and cognitive ability. Although we do not know the entry score needed to enter an academic focused secondary school, previous research on the 1958 cohort showed that the selective system saw increases in the probability of attending an academic focused school (grammar schools) from around the mid-point of distribution of the cognitive ability test, with the probability rising as test score increases (Pastore & Jones, 2019). It is around this point that we see in our diagram those in the selective system diverging from the comprehensive system in terms of age leaving education and highest qualification achieved. This suggests that schooling for those with higher cognitive ability scores is driving these education outcome differences between the systems. The results for the 1970 cohort are similar (see supplement 3).

### 4.5 Sensitivity analysis

Supplement 3 presents the results for the BMI at baseline as a negative control. It shows little evidence of any inequality by social class providing additional assurance that residual confounding may not be a large bias as BMI at baseline was not included in the confounder model.

Supplement 4 shows the class results in Fig. 2, Fig. 3 but using imputed outcomes. Both show little change to the main analysis.

In Supplement 5 we rerun the analysis with the inverse probability weights for adjusting confounding generated from an entropy balancing model that will achieve exact mean balance although it, as in this case, may do so with some observations having negative probabilities thus violating the positivity assumption that every observation should have a positive probability of receiving either of the “treatments.” In any case the results are not that changed, this is also the case in Supplement 6 where we use a generalized boosted model for inverse probability weight generation.

Supplement 7 shows that the pattern of results is not that changed when we exclude observations where the exposure was imputed.

Supplement 8 shows the results by school type at age 11 for the 1958 cohort rather than their school type at age 16. Results are similar, perhaps the largest change being that inequality in age left education is not so clearly higher in the selective system.

In summary, the overall conclusions were not changed by the sensitivity analysis.

## 5 Discussion

### 5.1 Main results

In an observational study of two British birth cohorts followed to middle age we found little evidence that health and wellbeing outcomes were different in the comprehensive compared to the selective school system both in terms of average outcomes and inequality in outcomes. This was despite there being differences in our education outcomes (on average slightly higher in the selective system) and social class inequality in education (lower in the comprehensive system) between the systems. Broadly, the results were consistent across the cohorts. We had originally hypothesised these differences in education outcomes could lead to health differences between systems in at least three ways. Firstly, through the direct effect of improvements in education on knowledge resources and in particular health literacy. As shown in Fig. 2 average education outcomes were slightly better in the selective system. Hence the idea that comprehensive system brought about changes in health literacy through improved average education is not well supported. Previous studies of education outcomes have tended to find that in the selective system average education outcomes are better for those attending academically focused schools compared to similar students in comprehensive schools but that comprehensive schools perform better for pupils who would have gone to vocationally focused schools in the selective system, so at the population level there is a small or no overall effect (Atkinson et al., 2006). Our exploratory analysis assessing the interaction between cognitive ability test score and system suggested, tentatively, that system differences in education emerged because of higher outcomes in those with higher ability scores in the selective system, who were most likely to attend an academic focused school (Fig. 5). Inequalities in education outcomes were reduced under the comprehensive system we found (see Fig. 3) but these seemed not to translate into reduced health inequalities. Similarly, recent UK research comparing outcomes in the selective system only for those who could have gone to an academic school, based on having a cognitive ability score near the cut off but some of whom did not obtain a place, found evidence of effects on education by school type but no effects on health (Butler et al., 2020; Clark and Royer, 2013; Pastore & Jones, 2019). While there was evidence of educational inequalities in BMI being lower in one cohort, across the other health outcomes there was little difference between the school systems whether we assessed inequality by education or social class. This suggests that the results for social class stratification broadly held when using a direct measure of education.

Our second possible mechanism through which the comprehensive system might have reduced health inequalities was by reducing inequalities in other social determinants of health. While we found in Fig. 4 destination social class inequalities by education were higher in the selective system this appeared not to translate into an effect on health inequalities. The inequalities in destination social class by education were large even in the comprehensive system, so even though reduced compared to the selective system the reduction may have been too small to impact health inequalities.

Our third mechanism, following the income inequality hypothesis, was the possibility that more equity in education and other social determinants of health would bring about greater social cohesion and thus improve population health and reduce health inequalities. While we observed reductions in inequality in education outcomes under the comprehensive system, this did not seem to translate into reductions in health inequalities. More directly, previous UK research found that the change to the comprehensive system did not improve indicators of social cohesion (Paterson, 2012).

In summary we found no consistent impact of the introduction of a comprehensive school system in Britain on adult health and health inequalities despite, in theory at least, the comprehensive system being more equitable. Overall, the equity impacts of a comprehensive system over a selective school system may be relatively minor. This is likely to be due to between-school and within-school differences which continue to exist in a comprehensive system, even though in a less visible way than in a selective system. There continue to be significant differentiation at the school level in the comprehensive system through geographical and socio-economic variation in catchment areas for example, as well as within-school differentiation of teaching by ability groupings and curriculum.

### 5.2 Strengths and limitations

Our study has strengths, including a clear aim of evaluating causal relationships (Hernán, 2018), pre-outcome registration of the analysis, use of representative data from two rich cohort studies, multiple imputation and weighting, a full range of measures of inequalities in health, a variety of outcomes and extensive sensitivity analysis.

In an observational study when the exposure of interest is not randomised, we need to be cognisant of the possibility of residual confounding and selection bias. In terms of residual confounding this is difficult to rule out, we did choose factors prior to secondary school such as region of residence, socio-economic background, parental education and aspirations for their child as well as the cognitive ability test scores that may impact whether they sought a particular school system (for example, seeking an academic school in the selective system). We were able to achieve good balance on these in our main propensity score and the two additional propensity score methods (one of which achieved “perfect” balance by design). We used the negative control of one of the outcomes (BMI) measured at the end of primary school. Despite not being included in the propensity score model it showed good balance as well.

Selection biases relates to how people are selected into the sample (Hernán & Robins, 2020). These include: volunteer bias - our samples were all births in one week with a high response rate; missing data bias-we used multiple imputation to mitigate this; and loss to follow-up - we used IPW to mitigate this as well as sensitivity analysis on multiple imputed outcomes.

Measurement error could also bias our results. In the 1958 cohort some schools were transitioning to the comprehensive system and so we conducted sensitivity analysis of the results to changing when we assessed school type (at age 11 or 16). We also compared results in 1958 to 1970 when the comprehensive system was more established which confirmed our overall findings.

Despite these limitations, our study adds to the small evidence base on the impact of comprehensive schooling on health inequalities.

## Funding

Economic and Social Research Council (ESRC Grant Reference ES/P009301/1). The funder had no role in the study, the writing of the article, or the decision to submit it for publication.

## CRediT author statement

**FP** Conceptualization, Methodology, Validation, Formal Analysis, Writing. **CI** Conceptualization, Methodology, Writing, Supervision, Funding acquisition.

## Ethics statement

Details of the ethics for the two cohort studies analysed can be found at https://cls.ucl.ac.uk/wp-content/uploads/2017/07/NCDS-Ethical-review-and-Consent-2014.pdf and https://cls.ucl.ac.uk/wp-content/uploads/2017/07/BCS70-ethical-review-and-consent-Shepherd-P-November-2012.pdf. Ethical approval was not required for the analysis in the paper as it was secondary analysis of anonymised data involving no primary data collection.

## Code and data availability

Code to replicate the analysis and the paper is available at https://github.com/frankpopham/comprehensive. The data itself is available to researchers from the UK data archive; we are not permitted to directly share the data but details of datasets used are in the code.

## Declaration of competing interest

None.
