# Supplementary material for: Does comprehensive education reduce health inequalities?
Source: SSM Popul Health. 2021 Jun 6;15:100834. doi: 10.1016/j.ssmph.2021.100834 (PMC8215301; doi:10.1016/j.ssmph.2021.100834)
Supplement: Multimedia component 1 [file mmc1.docx]

# Supplement 1

Firstly, we used multiple imputation (MI) to impute missing data on exposure and confounders. Secondly, we used inverse probability weights to account for the following post intervention: missing outcome data, death and emigration. We derived the IPW from models of the propensity of having missing data on the outcome or not as a function of the exposure and confounders. These weights are then multiplied by those for confounding. Our multiple imputation used chained equations (van Buuren and Groothuis-Oudshoorn, 2011) with the imputation model featuring the outcomes, exposure, confounders, and additional auxiliary variables (see Table S1_1) that are predictive of both missingness and the main variable (in our case the exposure) being imputed (Leyrat et al. 2017; Silverwood et al. 2020). We checked the convergence of the model using 10 iterations. We used the rule of thumb that the number of imputations should be at least the percentage of missingness (68 and 72 imputations for 1958 and 1970 cohort respectively) (White et al. 2011). As outlined in Tables S1_2 and S1_3 in missingness was less for each individual variable and overall the imputations did not greatly change variable distribution. We conducted separate imputations for each cohort. In line with recommendations all analysis was conducted on each imputation, with the results for the outcome models combined using Rubin’s rules (Leyrat et al. 2017).

Leyrat, C., Seaman, S.R., White, I.R., Douglas, I., Smeeth, L., Kim, J., Resche-Rigon, M., Carpenter, J.R., Williamson, E.J., 2017. Propensity score analysis with partially observed covariates: How should multiple imputation be used? Statistical Methods in Medical Research 28, 3–19. <https://doi.org/10.1177/0962280217713032>

Silverwood, R., Narayanan, M., Dodgeon, B., Ploubidis, G., 2020. Handling missing data in the National Child Development Study: User guide. UCL Centre for Longitudinal Studies, London, UK.

White, I.R., Royston, P., Wood, A.M., 2011. Multiple imputation using chained equations: Issues and guidance for practice. Statistics in Medicine 30, 377–399.

van Buuren, S., Groothuis-Oudshoorn, K. (2011). mice: Multivariate Imputation by Chained Equations in R. Journal of Statistical Software, 45(3), 1-67. URL <https://www.jstatsoft.org/v45/i03/>.

*Table S1_1 Auxiliary variables used in the multiple imputation*

| 1958 | 1970 |
| --- | --- |
| Four cognitive ability scales at age 7 ([Shepherd, 2012](#ref-shepherd2012))   - Southgate Group Reading Test - Copying designs - Drawing a man test - Problem arithmetic test   Region of residence at birth and age 7  Social class of parent at birth and age 7 | Four cognitive ability scales at age 5 ([Parsons, 2014](#ref-parsons2014))   - English Picture Vocabulary Test - Schonell Reading Test - Copying designs - Human Figure Drawing Test   Region of residence at birth and age 5  Social class of parent at birth and age 5 |

Table S1_2 Distribution of confounders and exposure before and after imputation 1958 cohort

|  | | Before MI | | After MI | |
| --- | --- | --- | --- | --- | --- |
|  |  | N | % | N | % |
| State primary school | No | 588 | 5.4 | 930 | 5.9 |
|  | Yes | 10,362 | 94.6 | 14,785 | 94.1 |
|  | Missing | 766 |  |  |  |
| Region of residence | North | 803 | 7.2 | 1,083 | 6.9 |
|  | North West | 1,364 | 12.3 | 2,021 | 12.9 |
|  | E & W.Riding | 947 | 8.5 | 1,316 | 8.4 |
|  | North Midlands | 866 | 7.8 | 1,221 | 7.8 |
|  | Midlands | 1,036 | 9.3 | 1,464 | 9.3 |
|  | East | 936 | 8.4 | 1,352 | 8.6 |
|  | South East | 1,787 | 16.1 | 2,773 | 17.6 |
|  | South | 664 | 6.0 | 988 | 6.3 |
|  | South West | 724 | 6.5 | 965 | 6.1 |
|  | Wales | 657 | 5.9 | 866 | 5.5 |
|  | Scotland | 1,342 | 12.1 | 1,665 | 10.6 |
|  | Missing | 590 |  |  |  |
| Sex | Male | 5,946 | 50.8 | 8,036 | 51.1 |
|  | Female | 5,770 | 49.2 | 7,678 | 48.9 |
| Age parent left education | 15 or below | 8,151 | 72.2 | 11,501 | 73.2 |
|  | 16 to 18 | 2,504 | 22.2 | 3,352 | 21.3 |
|  | 19 plus | 632 | 5.6 | 861 | 5.5 |
|  | Missing | 429 |  |  |  |
| Father's NSSEC | Higher managerial | 1,314 | 15.8 | 2,440 | 15.5 |
|  | Lower managerial | 364 | 4.4 | 686 | 4.4 |
|  | Intermediate | 1,789 | 21.6 | 3,382 | 21.5 |
|  | Routine | 4,824 | 58.2 | 9,206 | 58.6 |
|  | Missing | 3,425 |  |  |  |
| Parents interest in school rated by school | Very interested | 4,289 | 42.6 | 6,572 | 41.8 |
|  | Some interest | 4,287 | 42.6 | 6,733 | 42.8 |
|  | Little interest | 1,499 | 14.9 | 2,410 | 15.3 |
|  | Missing | 1,641 |  |  |  |
| Ethnicity | Asian | 12 | 0.1 | 30 | 0.2 |
|  | Black | 31 | 0.4 | 76 | 0.5 |
|  | Other | 48 | 0.6 | 93 | 0.6 |
|  | White | 8,376 | 98.9 | 15,515 | 98.7 |
|  | Missing | 3,249 |  |  |  |
| When parents hope will levave school | Leave at minimum age | 511 | 5.0 | 823 | 5.2 |
|  | Stay on longer | 7,755 | 76.5 | 11,868 | 75.5 |
|  | Don't know yet | 1,877 | 18.5 | 3,023 | 19.2 |
|  | Missing | 1,573 |  |  |  |
| Parents hope stays in education post school | Yes | 8,474 | 83.6 | 13,013 | 82.8 |
|  | No | 269 | 2.7 | 469 | 3.0 |
|  | Don't know yet | 1,398 | 13.8 | 2,232 | 14.2 |
|  | Missing | 1,575 |  |  |  |
| School system | Comprehensive | 7,112 | 60.7 | 9,428 | 60.0 |
|  | Selective | 4,604 | 39.3 | 6,287 | 40.0 |
| Cognitive ability | (mean) | 10,351 | 100.7 | 15,715 | 100.2 |

Table 1_3 Distribution of confounders and exposure before and after imputation 1970 cohort

|  | | Before MI | | After MI | |
| --- | --- | --- | --- | --- | --- |
|  |  | N | % | N | % |
| Region of residence | North | 610 | 7.1 | 950 | 6.1 |
|  | Yorks and Humberside | 835 | 9.8 | 1,457 | 9.3 |
|  | East Midlands | 660 | 7.7 | 1,120 | 7.1 |
|  | East Anglia | 347 | 4.1 | 540 | 3.4 |
|  | South East | 2,139 | 25.0 | 4,491 | 28.6 |
|  | South West | 698 | 8.2 | 1,185 | 7.5 |
|  | West Midlands | 892 | 10.4 | 1,606 | 10.2 |
|  | North West | 1,078 | 12.6 | 1,976 | 12.6 |
|  | Wales | 507 | 5.9 | 863 | 5.5 |
|  | Scotland | 779 | 9.1 | 1,512 | 9.6 |
|  | Missing | 525 |  |  |  |
| Sex | Male | 4,396 | 48.5 | 8,089 | 51.5 |
|  | Female | 4,674 | 51.5 | 7,610 | 48.5 |
| Age parent left education | 15 or below | 3,864 | 48.7 | 8,189 | 52.2 |
|  | 16 to 18 | 2,870 | 36.1 | 5,263 | 33.5 |
|  | 19plus | 1,206 | 15.2 | 2,248 | 14.3 |
|  | Missing | 1,130 |  |  |  |
| Father's NSSEC | Higher managerial | 1,625 | 22.4 | 3,041 | 19.4 |
|  | Lower managerial | 421 | 5.8 | 897 | 5.7 |
|  | Intermediate | 1,628 | 22.4 | 3,425 | 21.8 |
|  | Routine | 3,582 | 49.4 | 8,336 | 53.1 |
|  | Missing | 1,814 |  |  |  |
| Parents interest in school rated by school | Very interested | 4,006 | 60.0 | 8,584 | 54.7 |
|  | Some interest | 2,206 | 33.0 | 5,625 | 35.8 |
|  | Little interest | 464 | 7.0 | 1,491 | 9.5 |
|  | Missing | 2,394 |  |  |  |
| Ethnicity | European UK / Other | 7,540 | 97.7 | 15,144 | 96.5 |
|  | Indian / Pakistani / other Asian | 102 | 1.3 | 292 | 1.9 |
|  | Other | 15 | 0.2 | 48 | 0.3 |
|  | West Indian / African | 60 | 0.8 | 215 | 1.4 |
|  | Missing | 1,353 |  |  |  |
| When parents hope will levave school | 16 years old | 3,035 | 39.8 | 6,783 | 43.2 |
|  | 17 years old | 1,231 | 16.2 | 2,456 | 15.6 |
|  | 18 years old | 3,351 | 44.0 | 6,461 | 41.2 |
|  | Missing | 1,453 |  |  |  |
| Parents hope stays in education post school | Yes | 3,333 | 41.5 | 6,234 | 39.7 |
|  | No | 295 | 3.7 | 738 | 4.7 |
|  | Cannot say | 4,396 | 54.8 | 8,727 | 55.6 |
|  | Missing | 1,046 |  |  |  |
| School system | Comprehensive | 7,724 | 85.2 | 13,368 | 85.1 |
|  | Selective | 1,346 | 14.8 | 2,332 | 14.9 |
| State primary school | (mean) | 7,531 | 1.0 | 15,699 | 1.0 |
| Cognitive ability | (mean) | 7,270 | 101.9 | 15,699 | 100.0 |

# Supplement 2

### Distribution of cognitive ability test


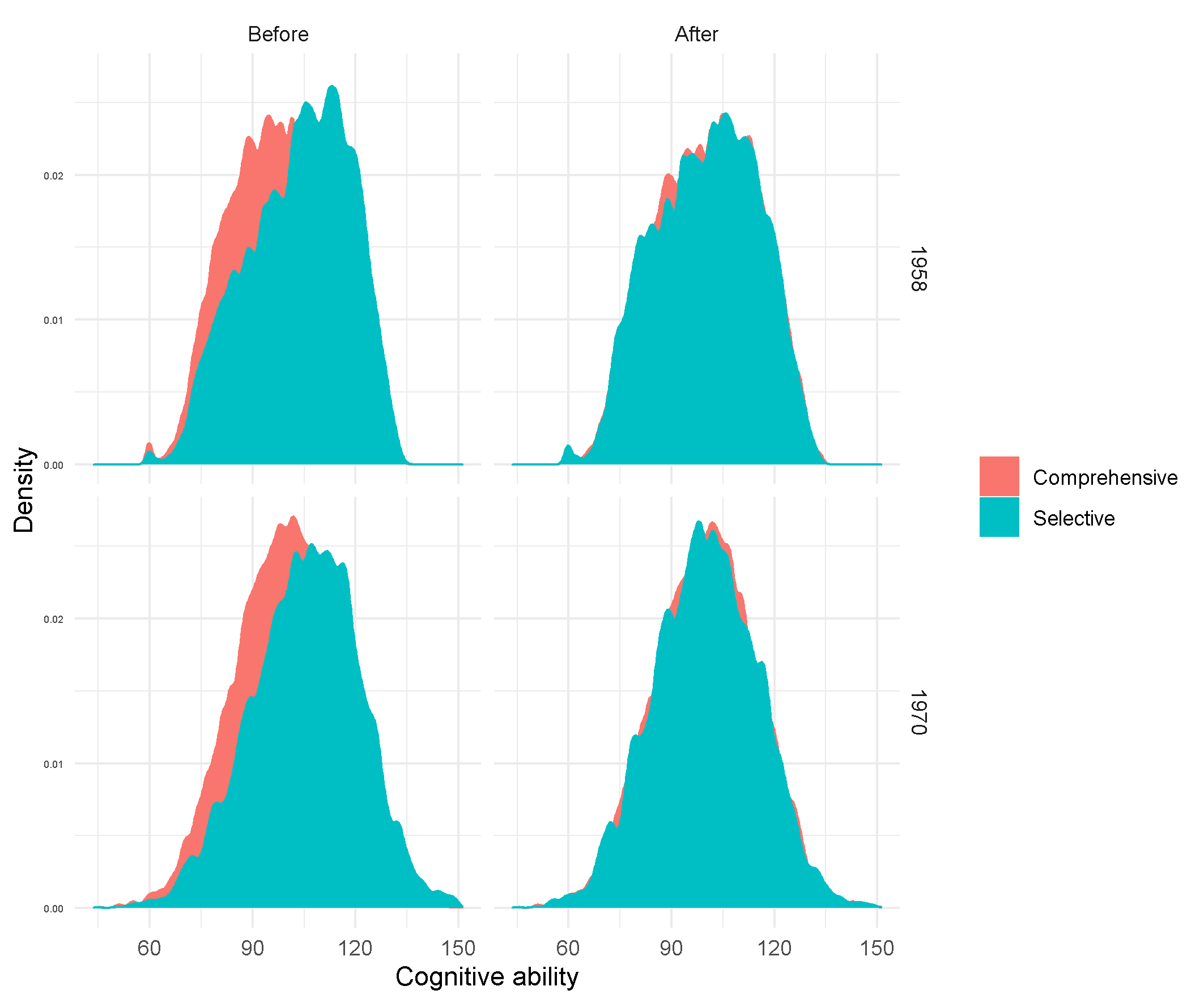


Figure 2_1Cognitive ability balance before and after weighting

# Supplement 3


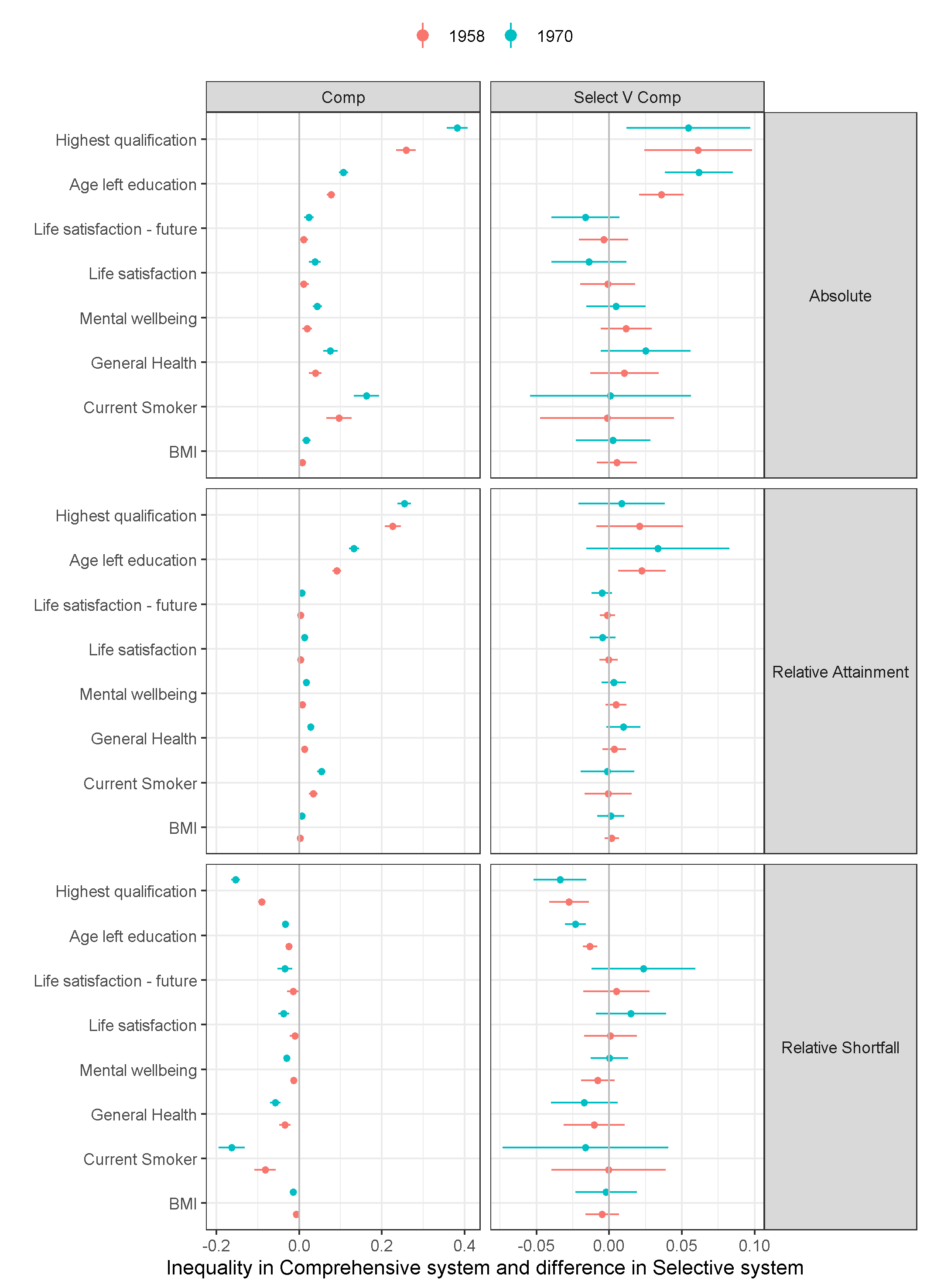


Figure S3_1 Destination class inequalities in outcomes by school system and cohort


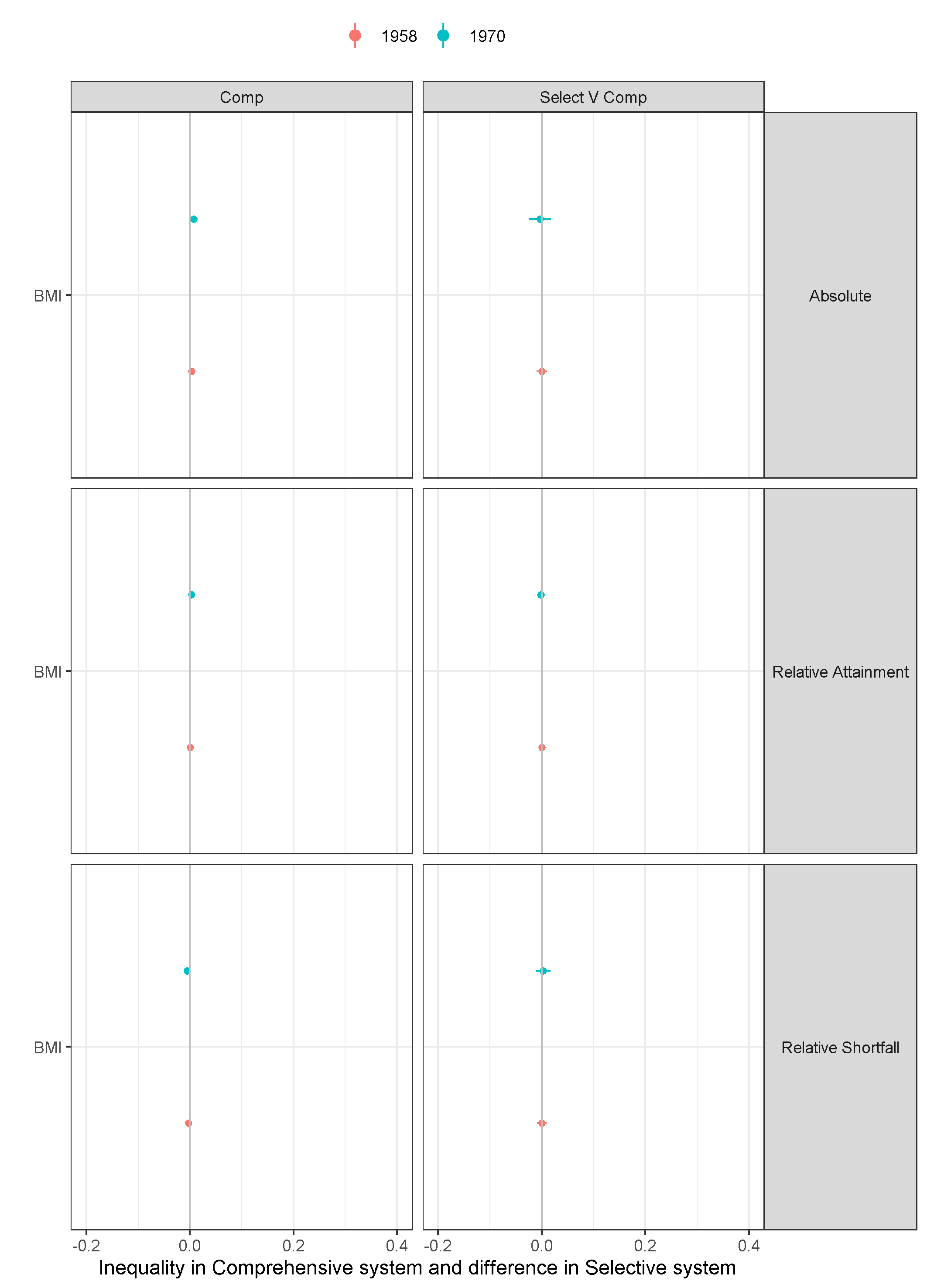


Figure S3_2 Negative control using BMI measured at age 10 or 11 (inequality by class of origin)


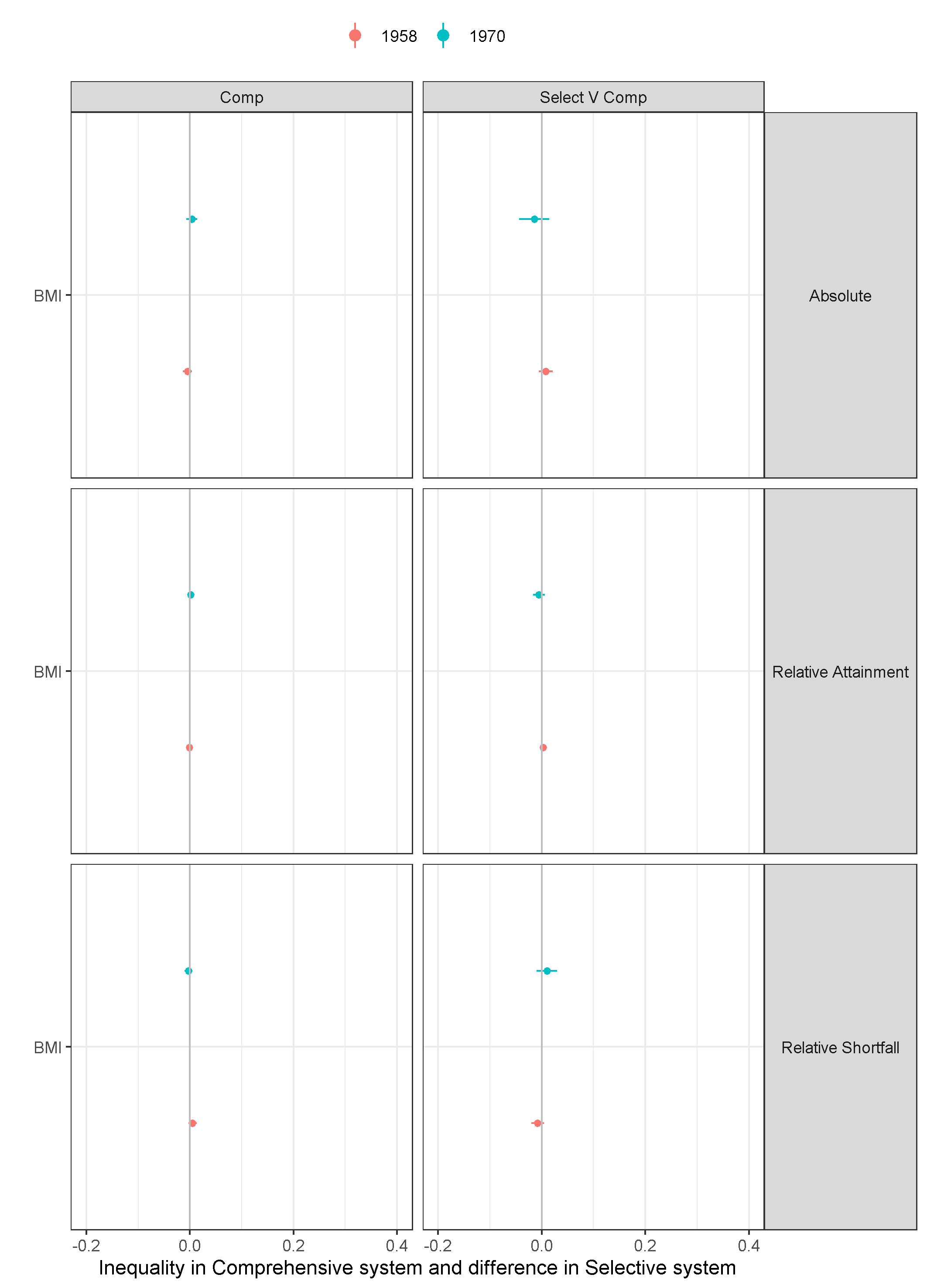


Figure S3_3 Negative control using BMI measured at age 10 or 11 (inequality by class of destination)


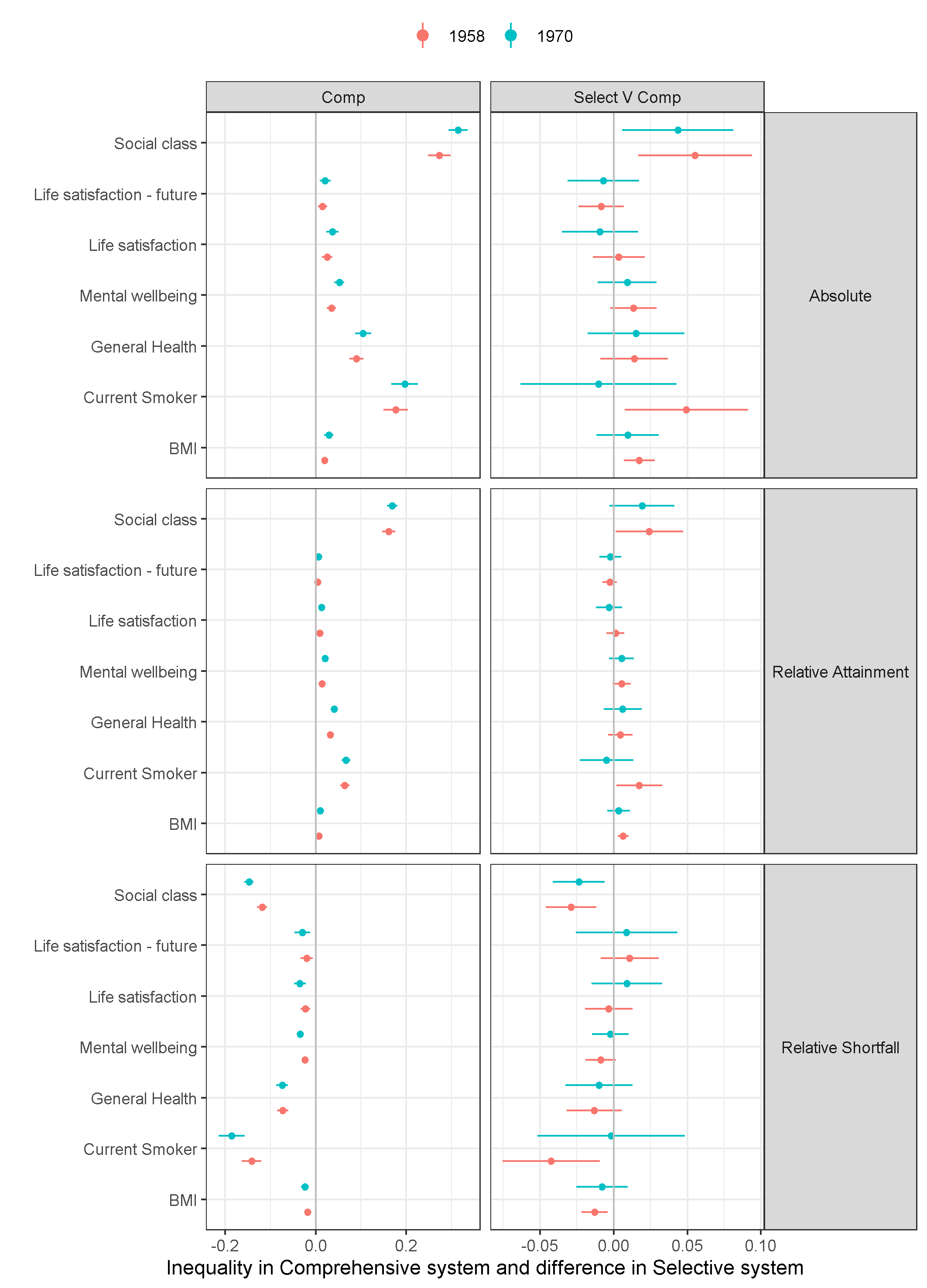


Figure S3_4 Education inequalities (highest qualification) in outcomes and social class by school system and cohort

# Supplement 4 Analysis repeated using imputed outcomes


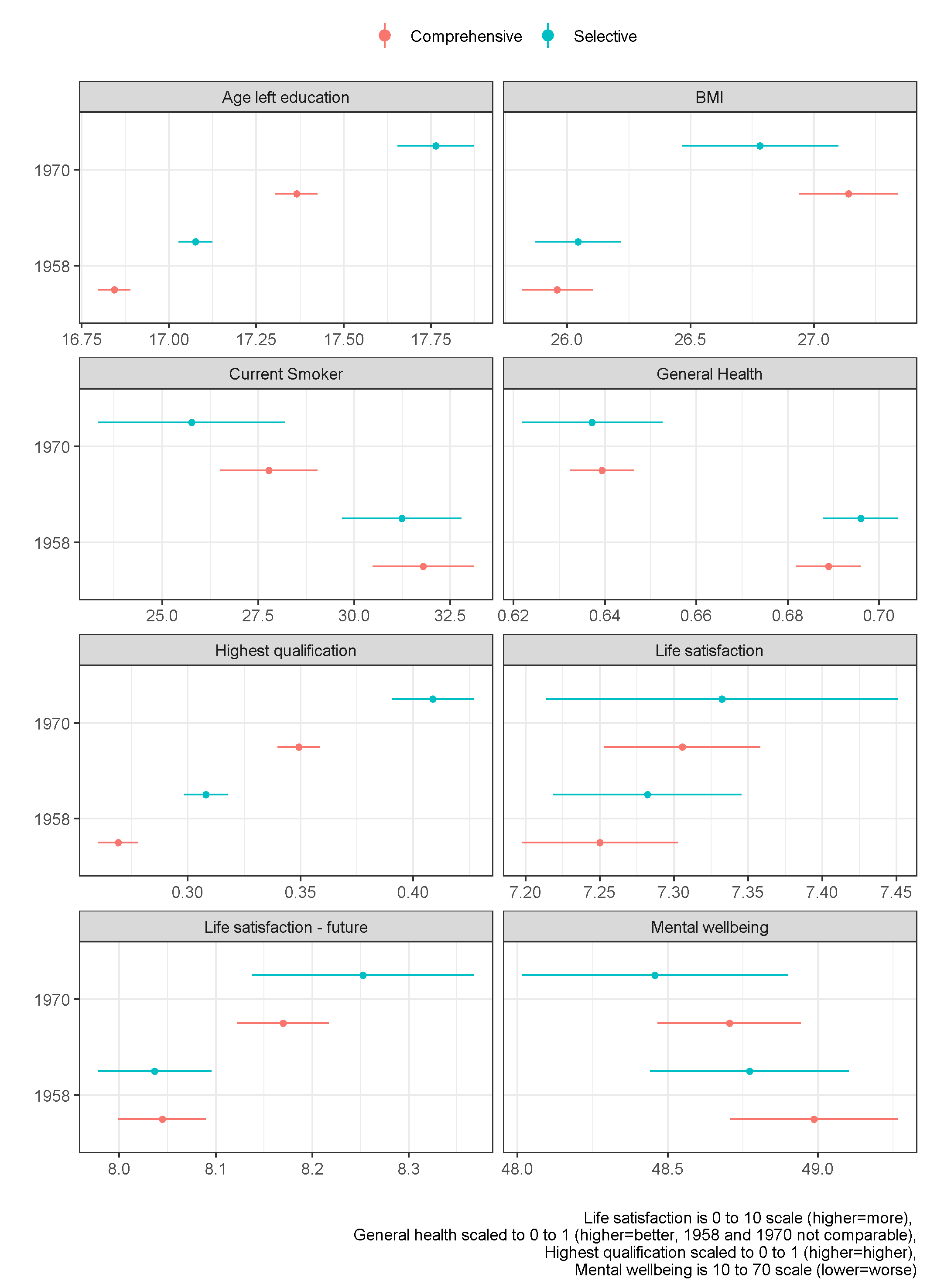


Figure S4_1 Mean outcomes by school system and cohort


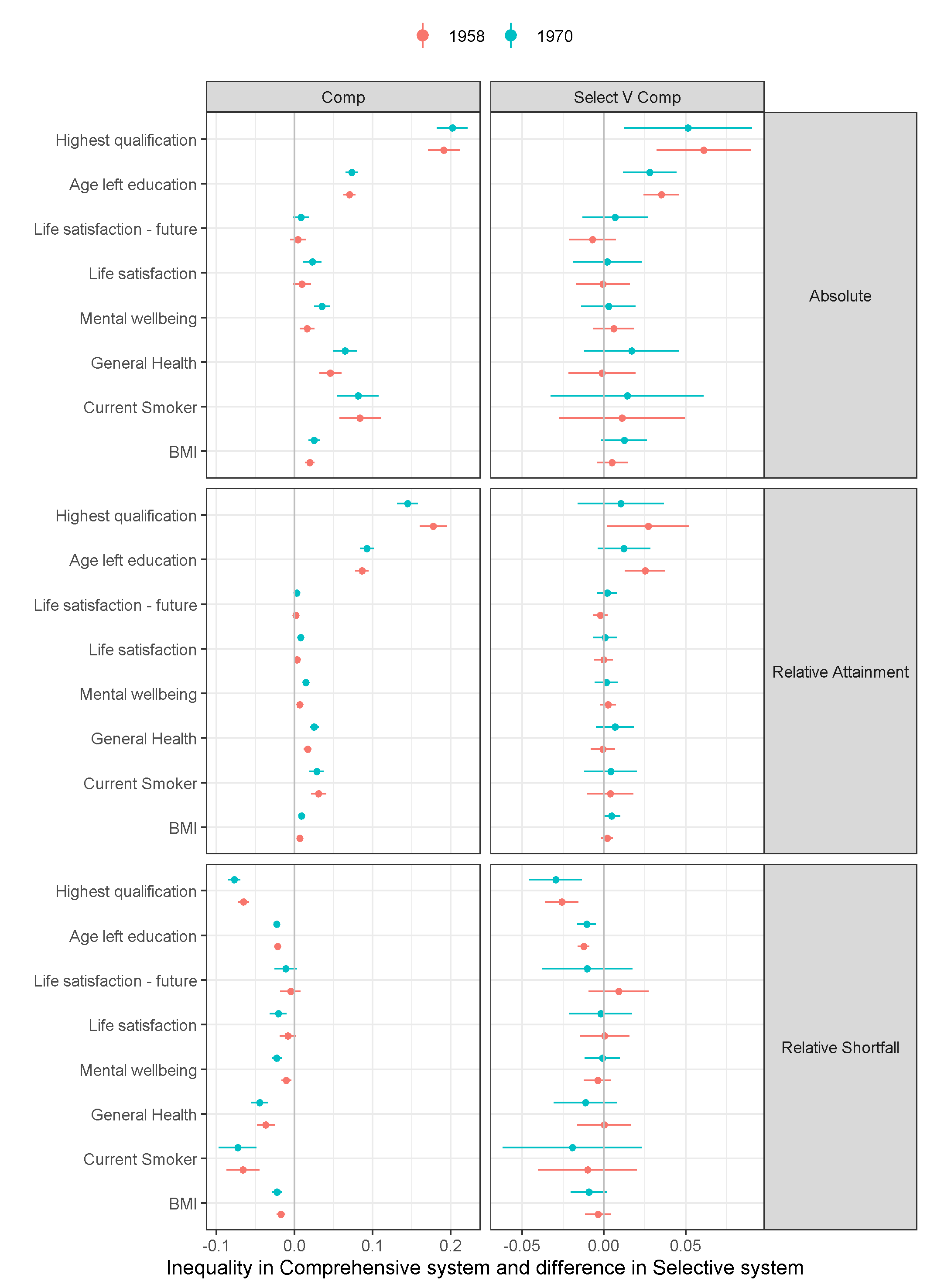


Figure S4_2 Origin class inequalities in outcomes by school system and cohort

# Supplement 5 Analysis repeated using entropy balancing

Table S5_1 Confounder balance before and after weighting

|  | | | Before | | After | |
| --- | --- | --- | --- | --- | --- | --- |
|  |  | All | Comp | Select | Comp | Select |
|  | **N** | **15,715** | **9,428** | **6,287** | **15,715** | **15,715** |
| Sex | Female | 48.9 | 48.5 | 49.4 | 48.9 | 48.9 |
|  | Male | 51.1 | 51.5 | 50.6 | 51.1 | 51.1 |
| Ethnicity | Asian | 0.2 | 0.2 | 0.1 | 0.2 | 0.2 |
|  | Black | 0.5 | 0.6 | 0.3 | 0.5 | 0.5 |
|  | Other | 0.6 | 0.7 | 0.5 | 0.6 | 0.6 |
|  | White | 98.7 | 98.5 | 99.1 | 98.7 | 98.7 |
| Cognitive ability | (mean) | 100.2 | 98.0 | 103.3 | 100.2 | 100.2 |
| Region of residence | E & W.Riding | 8.4 | 10.9 | 4.6 | 8.4 | 8.4 |
|  | East | 8.6 | 8.4 | 8.9 | 8.6 | 8.6 |
|  | Midlands | 9.3 | 8.4 | 10.7 | 9.3 | 9.3 |
|  | North | 6.9 | 6.8 | 7.0 | 6.9 | 6.9 |
|  | North Midlands | 7.8 | 7.0 | 8.9 | 7.8 | 7.8 |
|  | North West | 12.9 | 10.3 | 16.7 | 12.9 | 12.9 |
|  | Scotland | 10.6 | 15.9 | 2.6 | 10.6 | 10.6 |
|  | South | 6.3 | 4.9 | 8.4 | 6.3 | 6.3 |
|  | South East | 17.6 | 14.2 | 22.8 | 17.6 | 17.6 |
|  | South West | 6.1 | 5.6 | 6.9 | 6.1 | 6.1 |
|  | Wales | 5.5 | 7.5 | 2.5 | 5.5 | 5.5 |
| Age parent left education | 15 or below | 73.2 | 78.8 | 64.8 | 73.2 | 73.2 |
|  | 16 to 18 | 21.3 | 17.9 | 26.5 | 21.3 | 21.3 |
|  | 19 plus | 5.5 | 3.3 | 8.7 | 5.5 | 5.5 |
| When parents hope will leave school | Don't know yet | 19.2 | 20.4 | 17.4 | 19.2 | 19.2 |
|  | Leave at minimum age | 5.2 | 5.7 | 4.6 | 5.2 | 5.2 |
|  | Stay on longer | 75.5 | 73.9 | 78.0 | 75.5 | 75.5 |
| Parents hope stays in education post school | Don't know yet | 14.2 | 15.4 | 12.4 | 14.2 | 14.2 |
|  | No | 3.0 | 3.1 | 2.9 | 3.0 | 3.0 |
|  | Yes | 82.8 | 81.5 | 84.7 | 82.8 | 82.8 |
| Parents interest in school rated by school | Little interest | 15.3 | 17.4 | 12.2 | 15.3 | 15.3 |
|  | Some interest | 42.8 | 45.7 | 38.5 | 42.8 | 42.8 |
|  | Very interested | 41.8 | 36.9 | 49.2 | 41.8 | 41.8 |
| State primary school | No | 5.9 | 3.8 | 9.2 | 5.9 | 5.9 |
|  | Yes | 94.1 | 96.2 | 90.8 | 94.1 | 94.1 |
| Father's NSSEC | Higher managerial | 15.5 | 11.6 | 21.4 | 15.5 | 15.5 |
|  | Intermediate | 21.5 | 21.3 | 21.9 | 21.5 | 21.5 |
|  | Lower managerial | 4.4 | 3.5 | 5.7 | 4.4 | 4.4 |
|  | Routine | 58.6 | 63.7 | 51.0 | 58.6 | 58.6 |
|  | N | 15,699 | 13,368 | 2,332 | 15,699 | 15,699 |
| **Sex** | **Female** | **48.5** | **48.4** | **48.8** | **48.5** | **48.5** |
|  | Male | 51.5 | 51.6 | 51.2 | 51.5 | 51.5 |
| Ethnicity | European UK / Other | 96.5 | 96.4 | 97.1 | 96.5 | 96.5 |
|  | Indian / Pakistani / other Asian | 1.9 | 2.0 | 1.2 | 1.9 | 1.9 |
|  | Other | 0.3 | 0.3 | 0.1 | 0.3 | 0.3 |
|  | West Indian / African | 1.4 | 1.3 | 1.5 | 1.4 | 1.4 |
| Cognitive ability | (mean) | 100.0 | 99.0 | 105.5 | 100.0 | 100.0 |
| Region of residence | East Anglia | 3.4 | 3.6 | 2.3 | 3.4 | 3.4 |
|  | East Midlands | 7.1 | 7.3 | 6.1 | 7.1 | 7.1 |
|  | North | 6.1 | 6.7 | 2.4 | 6.1 | 6.1 |
|  | North West | 12.6 | 12.9 | 10.6 | 12.6 | 12.6 |
|  | Scotland | 9.6 | 9.5 | 10.6 | 9.6 | 9.6 |
|  | South East | 28.6 | 26.2 | 42.6 | 28.6 | 28.6 |
|  | South West | 7.5 | 7.2 | 9.8 | 7.5 | 7.5 |
|  | Wales | 5.5 | 5.9 | 2.9 | 5.5 | 5.5 |
|  | West Midlands | 10.2 | 10.8 | 7.0 | 10.2 | 10.2 |
|  | Yorks and Humberside | 9.3 | 9.9 | 5.7 | 9.3 | 9.3 |
| Age parent left education | 15 or below | 52.2 | 54.7 | 37.7 | 52.2 | 52.2 |
|  | 16 to 18 | 33.5 | 33.1 | 36.1 | 33.5 | 33.5 |
|  | 19plus | 14.3 | 12.2 | 26.2 | 14.3 | 14.3 |
| When parents hope will leave school | 16 years old | 43.2 | 45.7 | 28.6 | 43.2 | 43.2 |
|  | 17 years old | 15.6 | 15.9 | 14.4 | 15.6 | 15.6 |
|  | 18 years old | 41.2 | 38.4 | 57.0 | 41.2 | 41.2 |
| Parents hope stays in education post school | Cannot say | 55.6 | 57.8 | 42.9 | 55.6 | 55.6 |
|  | No | 4.7 | 5.0 | 2.9 | 4.7 | 4.7 |
|  | Yes | 39.7 | 37.2 | 54.2 | 39.7 | 39.7 |
| Parents interest in school rated by school | Little interest | 9.5 | 10.0 | 6.5 | 9.5 | 9.5 |
|  | Some interest | 35.8 | 37.3 | 27.2 | 35.8 | 35.8 |
|  | Very interested | 54.7 | 52.7 | 66.3 | 54.7 | 54.7 |
| State primary school | No | 3.7 | 1.5 | 16.4 | 3.7 | 3.7 |
|  | Yes | 96.3 | 98.5 | 83.6 | 96.3 | 96.3 |
| Father's NSSEC | Higher managerial | 19.4 | 17.2 | 32.0 | 19.4 | 19.4 |
|  | Intermediate | 21.8 | 22.0 | 20.8 | 21.8 | 21.8 |
|  | Lower managerial | 5.7 | 5.4 | 7.3 | 5.7 | 5.7 |
|  | Routine | 53.1 | 55.4 | 39.9 | 53.1 | 53.1 |


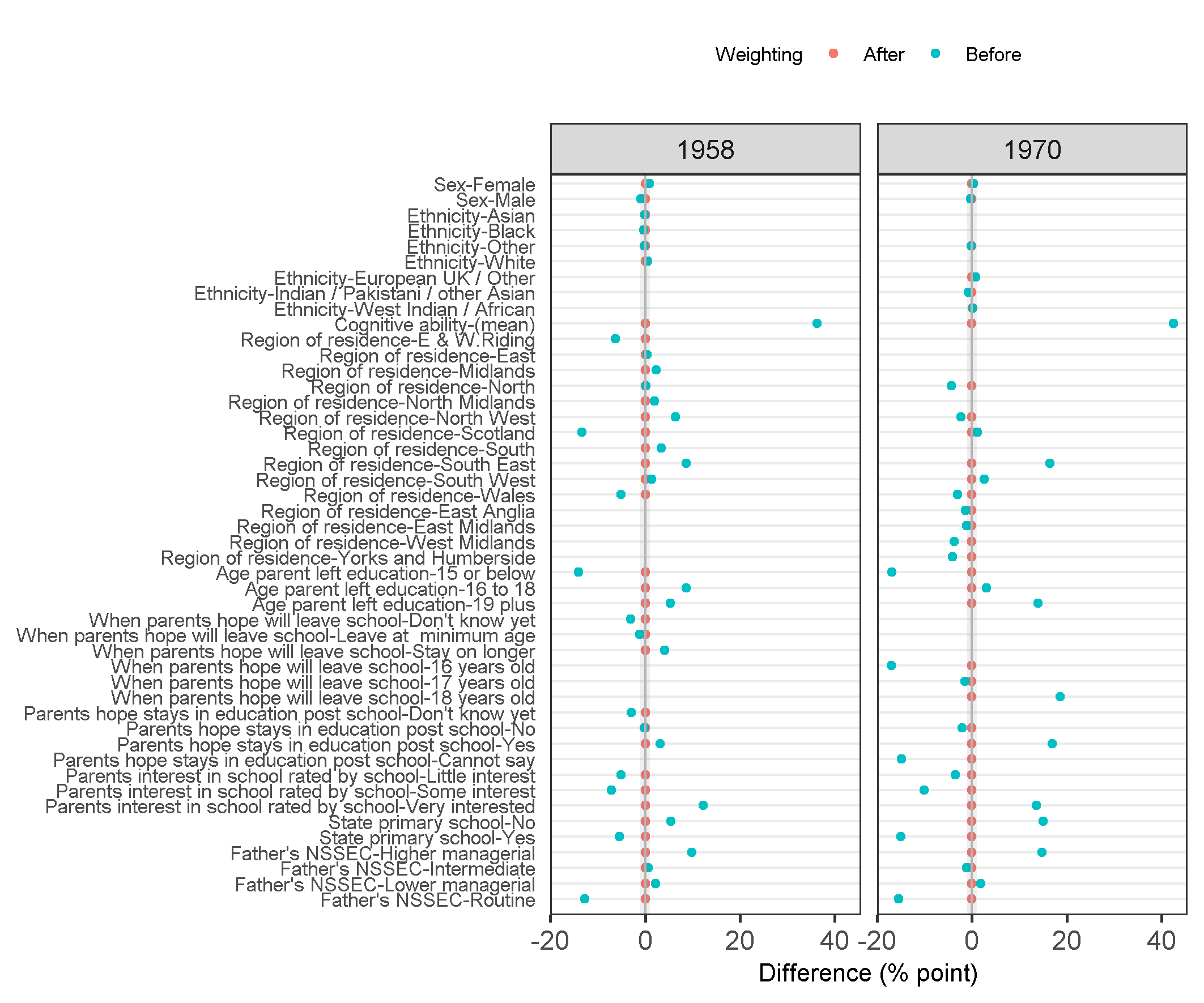


Figures5_1 Confounder balance before and after weighting


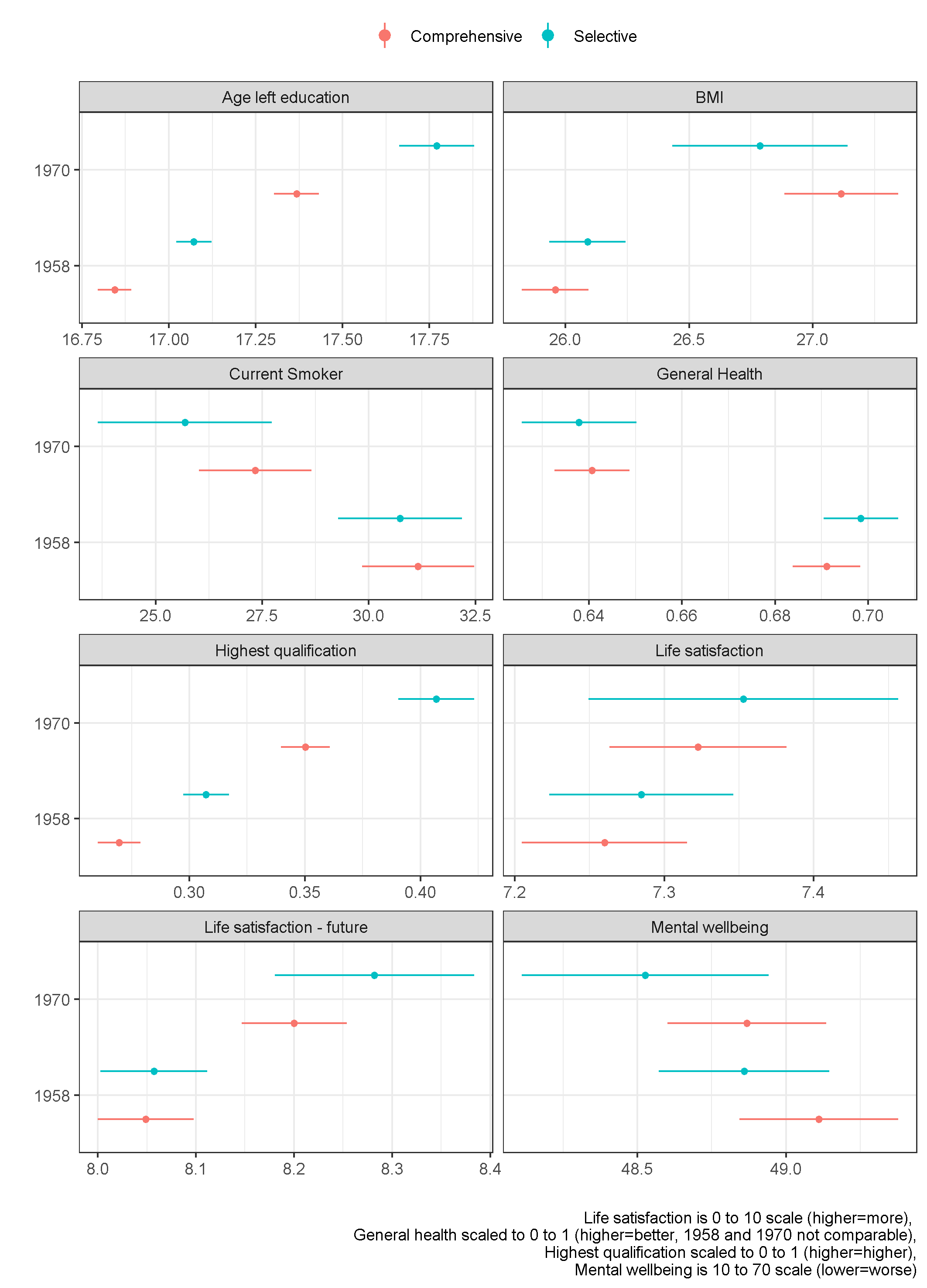


Figure S5_2 Mean outcomes by school system and cohort


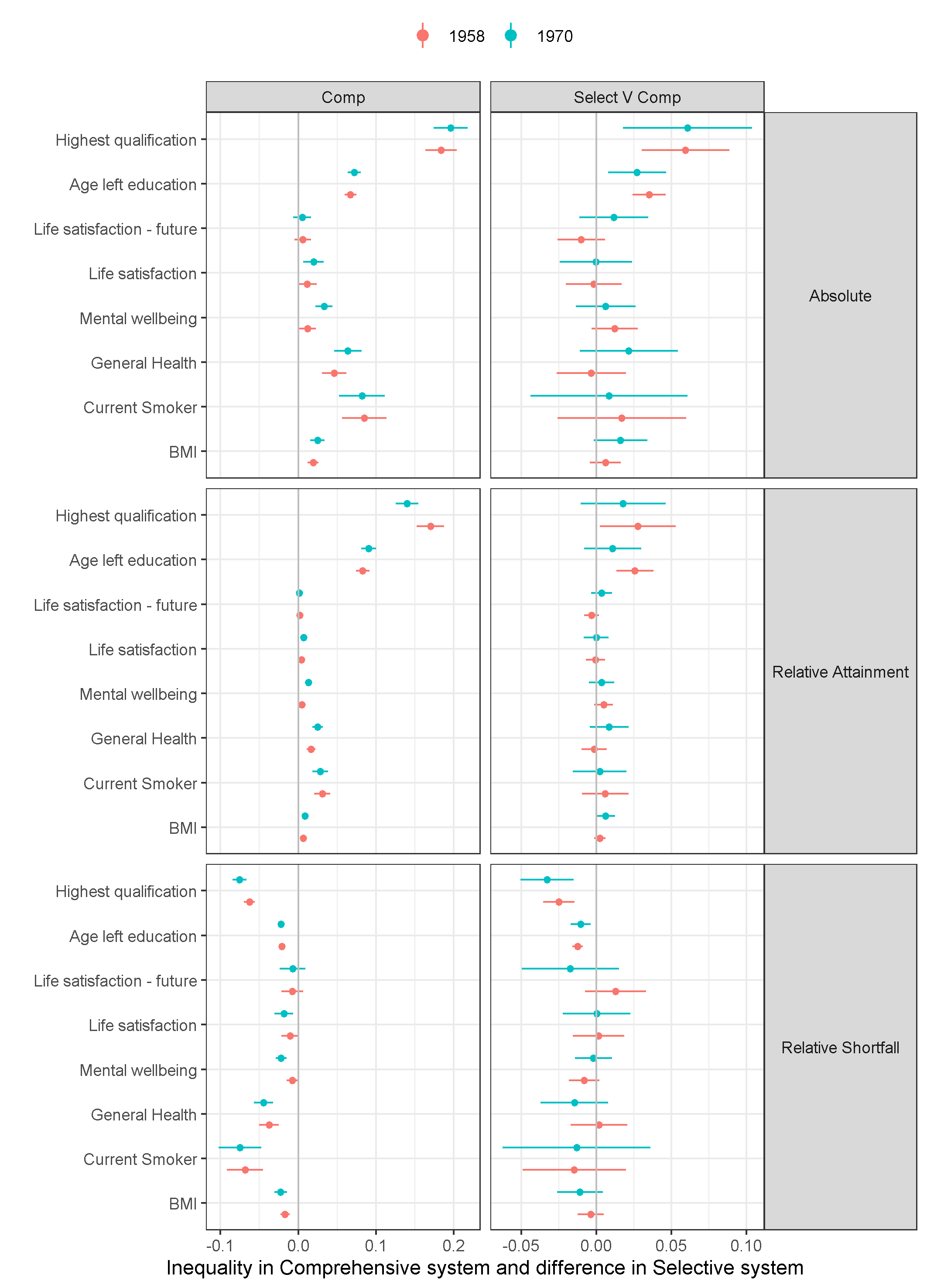


Figure S5_3 Origin class inequalities in outcomes by school system and cohort


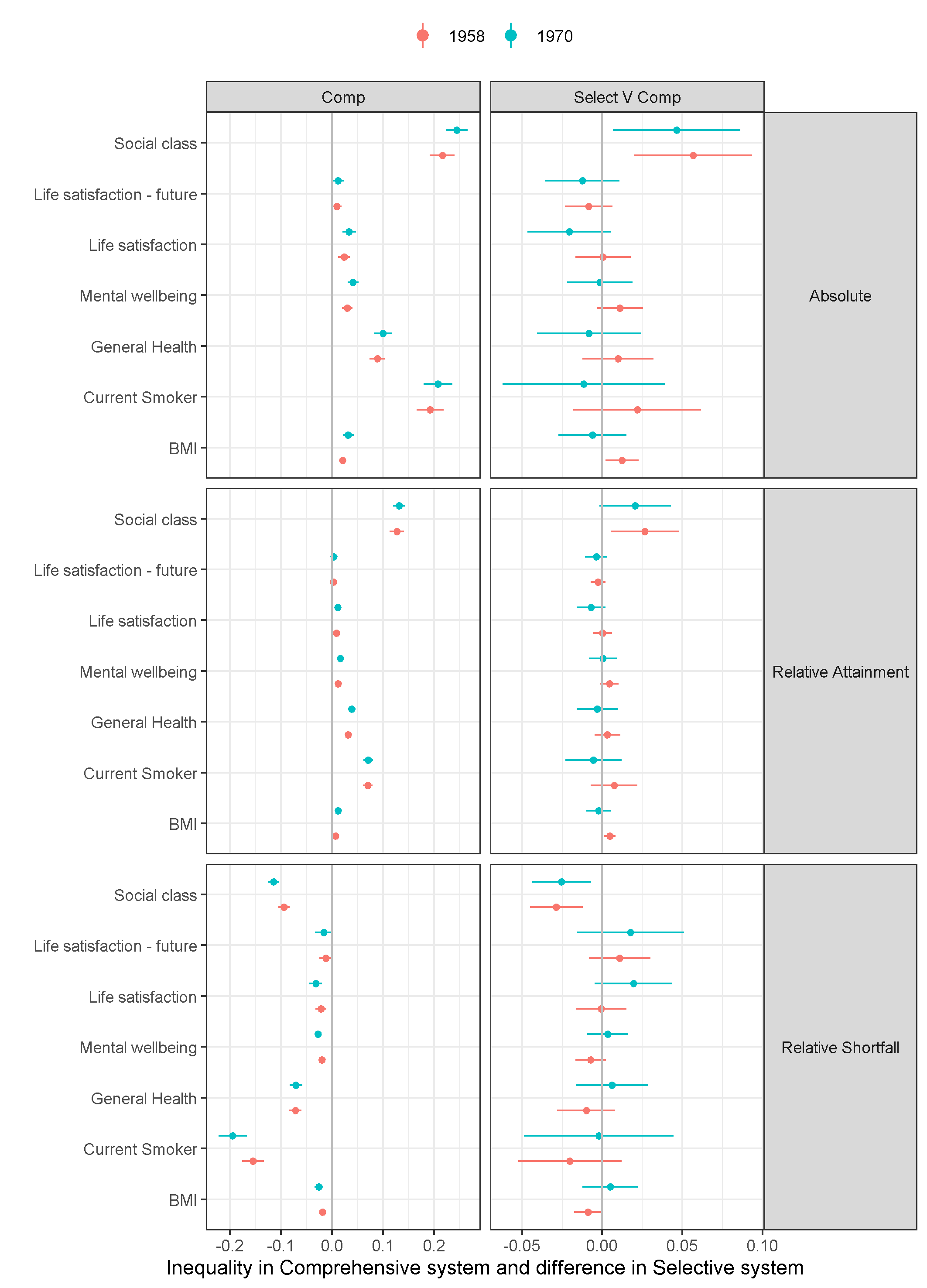


Figure S5_4 Education inequalities (age left education) in outcomes and social class by school system and cohort


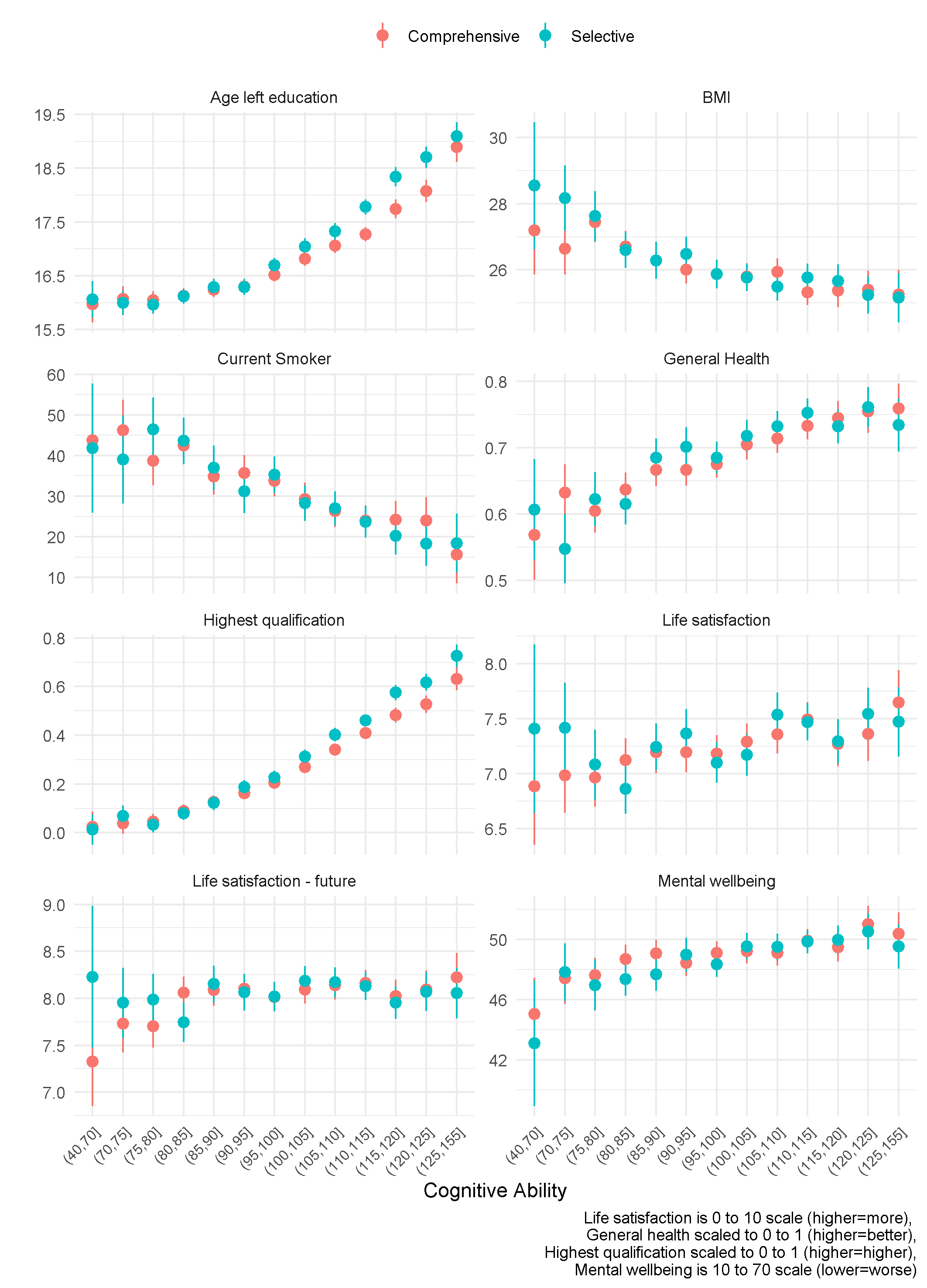


Figure S5_5 Mean of outcomes by cognitive ability and school system 1958 cohort

# Supplement 6 Analysis repeated using gbm

Table 6_1 Confounder balance before and after weighting

|  | | | Before | | After | |
| --- | --- | --- | --- | --- | --- | --- |
|  |  | All | Comp | Select | Comp | Select |
|  | **N** | **15,715** | **9,428** | **6,287** | **15,489** | **15,196** |
| Sex | Female | 48.9 | 48.5 | 49.4 | 48.9 | 48.4 |
|  | Male | 51.1 | 51.5 | 50.6 | 51.1 | 51.6 |
| Ethnicity | Asian | 0.2 | 0.2 | 0.1 | 0.2 | 0.1 |
|  | Black | 0.5 | 0.6 | 0.3 | 0.5 | 0.4 |
|  | Other | 0.6 | 0.7 | 0.5 | 0.6 | 0.5 |
|  | White | 98.7 | 98.5 | 99.1 | 98.8 | 98.9 |
| Cognitive ability | (mean) | 100.2 | 98.0 | 103.3 | 100.0 | 100.4 |
| Region of residence | E & W.Riding | 8.4 | 10.9 | 4.6 | 8.5 | 8.3 |
|  | East | 8.6 | 8.4 | 8.9 | 8.6 | 8.7 |
|  | Midlands | 9.3 | 8.4 | 10.7 | 9.3 | 9.5 |
|  | North | 6.9 | 6.8 | 7.0 | 6.9 | 7.0 |
|  | North Midlands | 7.8 | 7.0 | 8.9 | 7.8 | 8.0 |
|  | North West | 12.9 | 10.3 | 16.7 | 12.7 | 13.2 |
|  | Scotland | 10.6 | 15.9 | 2.6 | 10.7 | 9.5 |
|  | South | 6.3 | 4.9 | 8.4 | 6.2 | 6.4 |
|  | South East | 17.6 | 14.2 | 22.8 | 17.5 | 18.0 |
|  | South West | 6.1 | 5.6 | 6.9 | 6.1 | 6.2 |
|  | Wales | 5.5 | 7.5 | 2.5 | 5.6 | 5.2 |
| Age parent left education | 15 or below | 73.2 | 78.8 | 64.8 | 73.6 | 73.1 |
|  | 16 to 18 | 21.3 | 17.9 | 26.5 | 21.1 | 21.3 |
|  | 19 plus | 5.5 | 3.3 | 8.7 | 5.2 | 5.6 |
| When parents hope will leave school | Don't know yet | 19.2 | 20.4 | 17.4 | 19.2 | 19.4 |
|  | Leave at minimum age | 5.2 | 5.7 | 4.6 | 5.2 | 5.2 |
|  | Stay on longer | 75.5 | 73.9 | 78.0 | 75.6 | 75.4 |
| Parents hope stays in education post school | Don't know yet | 14.2 | 15.4 | 12.4 | 14.3 | 14.1 |
|  | No | 3.0 | 3.1 | 2.9 | 3.0 | 3.0 |
|  | Yes | 82.8 | 81.5 | 84.7 | 82.8 | 82.9 |
| Parents interest in school rated by school | Little interest | 15.3 | 17.4 | 12.2 | 15.4 | 15.1 |
|  | Some interest | 42.8 | 45.7 | 38.5 | 43.0 | 42.4 |
|  | Very interested | 41.8 | 36.9 | 49.2 | 41.6 | 42.5 |
| State primary school | No | 5.9 | 3.8 | 9.2 | 5.6 | 5.9 |
|  | Yes | 94.1 | 96.2 | 90.8 | 94.4 | 94.1 |
| Father's NSSEC | Higher managerial | 15.5 | 11.6 | 21.4 | 15.2 | 15.8 |
|  | Intermediate | 21.5 | 21.3 | 21.9 | 21.6 | 21.4 |
|  | Lower managerial | 4.4 | 3.5 | 5.7 | 4.2 | 4.4 |
|  | Routine | 58.6 | 63.7 | 51.0 | 59.0 | 58.4 |
|  | N | 15,699 | 13,368 | 2,332 | 15,570 | 14,155 |
| **Sex** | **Female** | **48.5** | **48.4** | **48.8** | **48.5** | **49.1** |
|  | Male | 51.5 | 51.6 | 51.2 | 51.5 | 50.9 |
| Ethnicity | European UK / Other | 96.5 | 96.4 | 97.1 | 96.5 | 97.1 |
|  | Indian / Pakistani / other Asian | 1.9 | 2.0 | 1.2 | 1.9 | 1.5 |
|  | Other | 0.3 | 0.3 | 0.1 | 0.3 | 0.1 |
|  | West Indian / African | 1.4 | 1.3 | 1.5 | 1.4 | 1.3 |
| Cognitive ability | (mean) | 100.0 | 99.0 | 105.5 | 99.9 | 100.3 |
| Region of residence | East Anglia | 3.4 | 3.6 | 2.3 | 3.4 | 2.9 |
|  | East Midlands | 7.1 | 7.3 | 6.1 | 7.2 | 7.5 |
|  | North | 6.1 | 6.7 | 2.4 | 6.1 | 4.9 |
|  | North West | 12.6 | 12.9 | 10.6 | 12.6 | 12.8 |
|  | Scotland | 9.6 | 9.5 | 10.6 | 9.6 | 10.4 |
|  | South East | 28.6 | 26.2 | 42.6 | 28.4 | 30.3 |
|  | South West | 7.5 | 7.2 | 9.8 | 7.5 | 7.9 |
|  | Wales | 5.5 | 5.9 | 2.9 | 5.5 | 5.0 |
|  | West Midlands | 10.2 | 10.8 | 7.0 | 10.3 | 9.7 |
|  | Yorks and Humberside | 9.3 | 9.9 | 5.7 | 9.3 | 8.6 |
| Age parent left education | 15 or below | 52.2 | 54.7 | 37.7 | 52.5 | 51.3 |
|  | 16 to 18 | 33.5 | 33.1 | 36.1 | 33.5 | 34.1 |
|  | 19plus | 14.3 | 12.2 | 26.2 | 14.0 | 14.5 |
| When parents hope will leave school | 16 years old | 43.2 | 45.7 | 28.6 | 43.5 | 41.9 |
|  | 17 years old | 15.6 | 15.9 | 14.4 | 15.7 | 16.5 |
|  | 18 years old | 41.2 | 38.4 | 57.0 | 40.8 | 41.7 |
| Parents hope stays in education post school | Cannot say | 55.6 | 57.8 | 42.9 | 55.9 | 54.9 |
|  | No | 4.7 | 5.0 | 2.9 | 4.7 | 4.4 |
|  | Yes | 39.7 | 37.2 | 54.2 | 39.4 | 40.6 |
| Parents interest in school rated by school | Little interest | 9.5 | 10.0 | 6.5 | 9.5 | 9.2 |
|  | Some interest | 35.8 | 37.3 | 27.2 | 36.1 | 35.3 |
|  | Very interested | 54.7 | 52.7 | 66.3 | 54.4 | 55.5 |
| State primary school | No | 3.7 | 1.5 | 16.4 | 3.2 | 3.9 |
|  | Yes | 96.3 | 98.5 | 83.6 | 96.8 | 96.1 |
| Father's NSSEC | Higher managerial | 19.4 | 17.2 | 32.0 | 19.1 | 20.2 |
|  | Intermediate | 21.8 | 22.0 | 20.8 | 21.9 | 22.3 |
|  | Lower managerial | 5.7 | 5.4 | 7.3 | 5.6 | 5.4 |
|  | Routine | 53.1 | 55.4 | 39.9 | 53.4 | 52.1 |


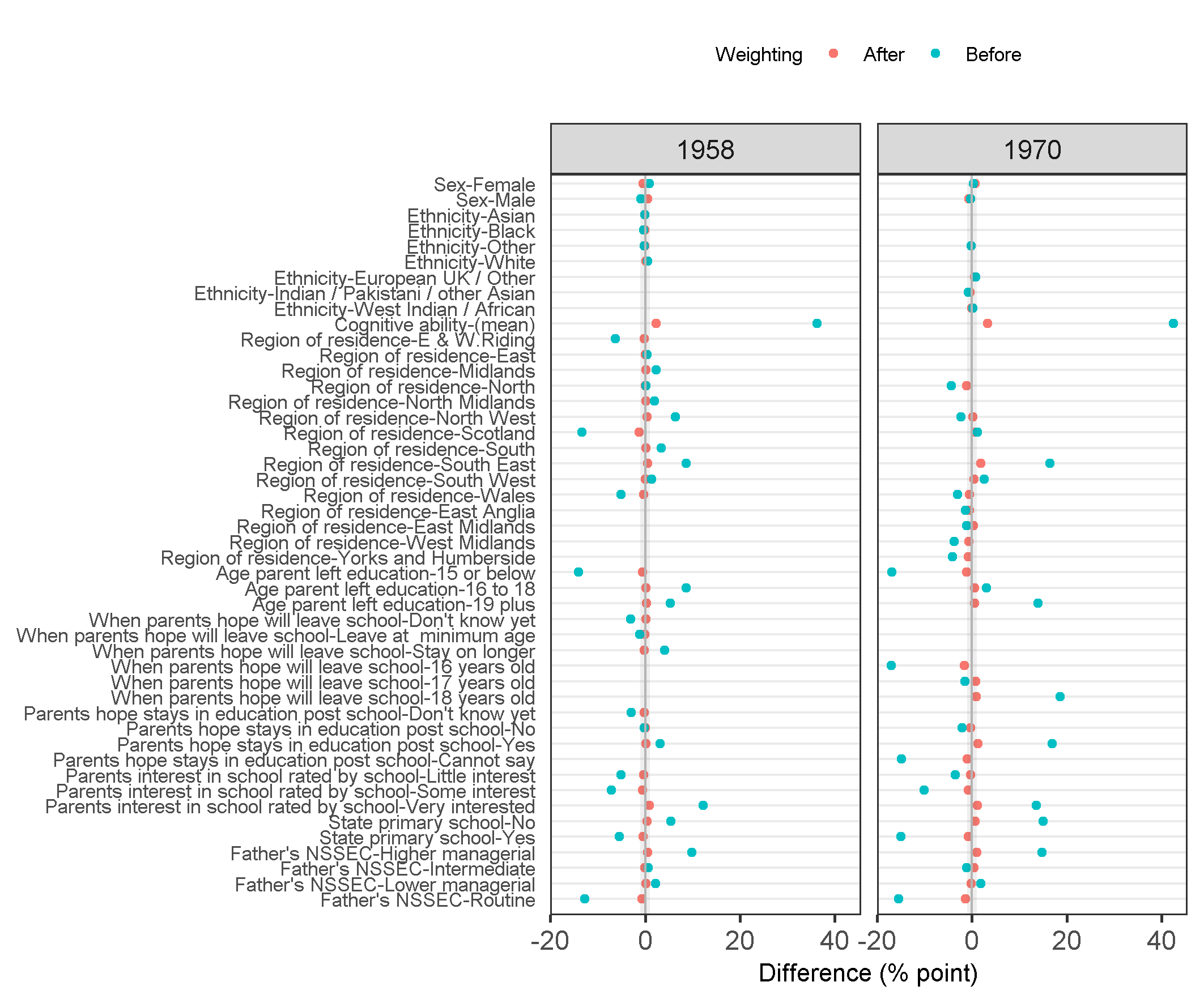


Figure S6_1 Confounder balance before and after weighting


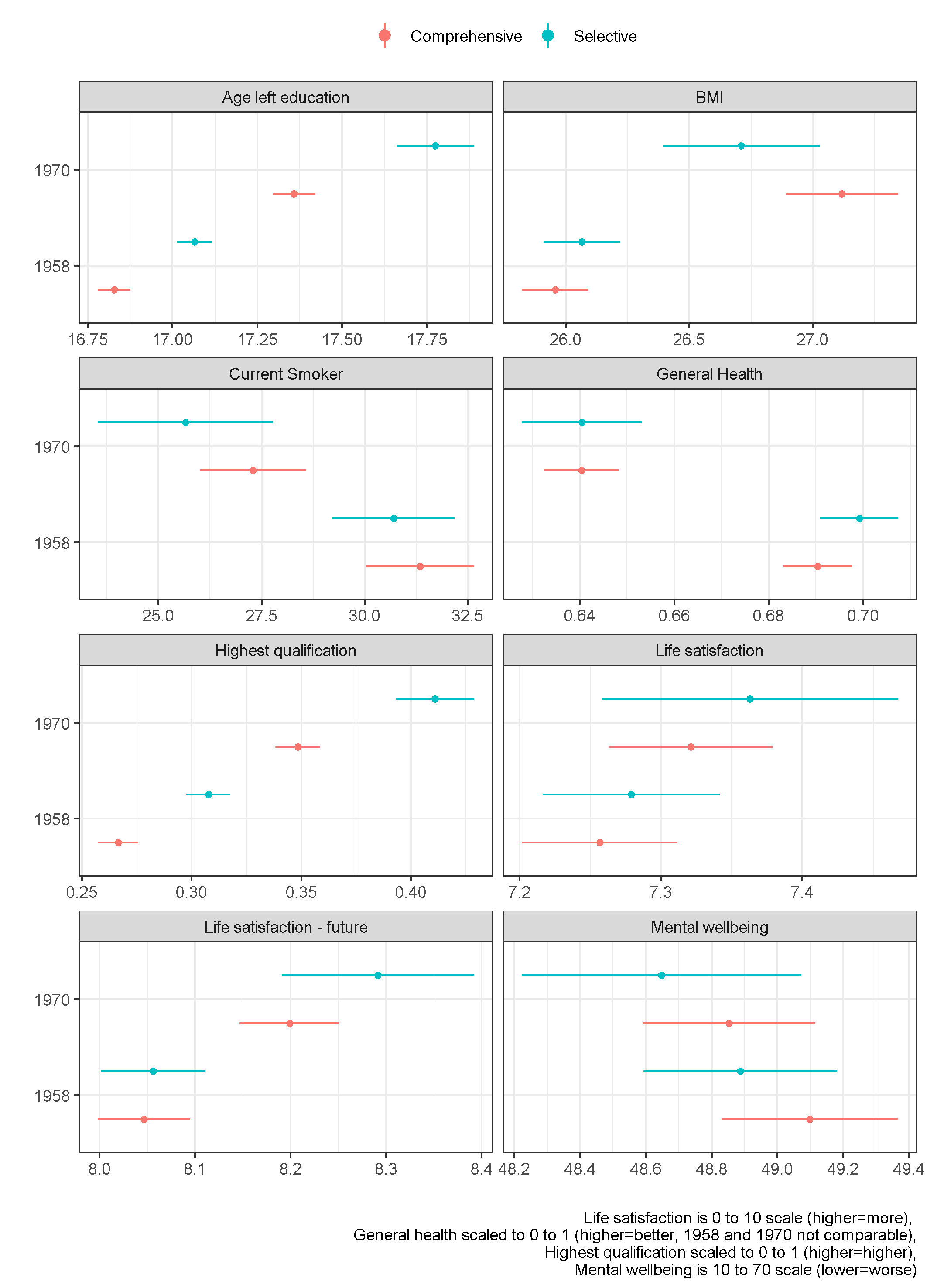


Figure S6_2 Mean outcomes by school system and cohort


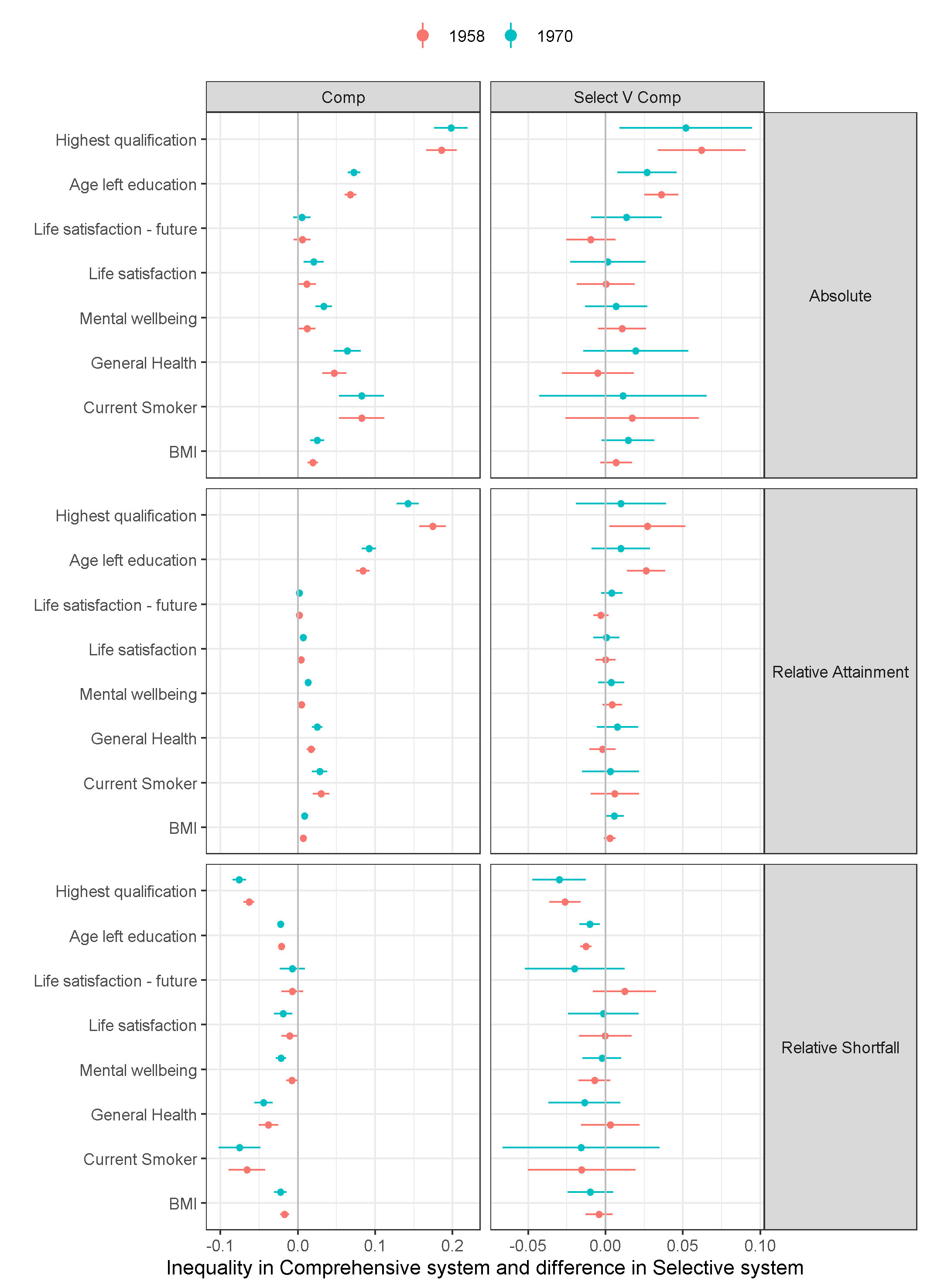


Figure S6_3 Origin class inequalities in outcomes by school system and cohort


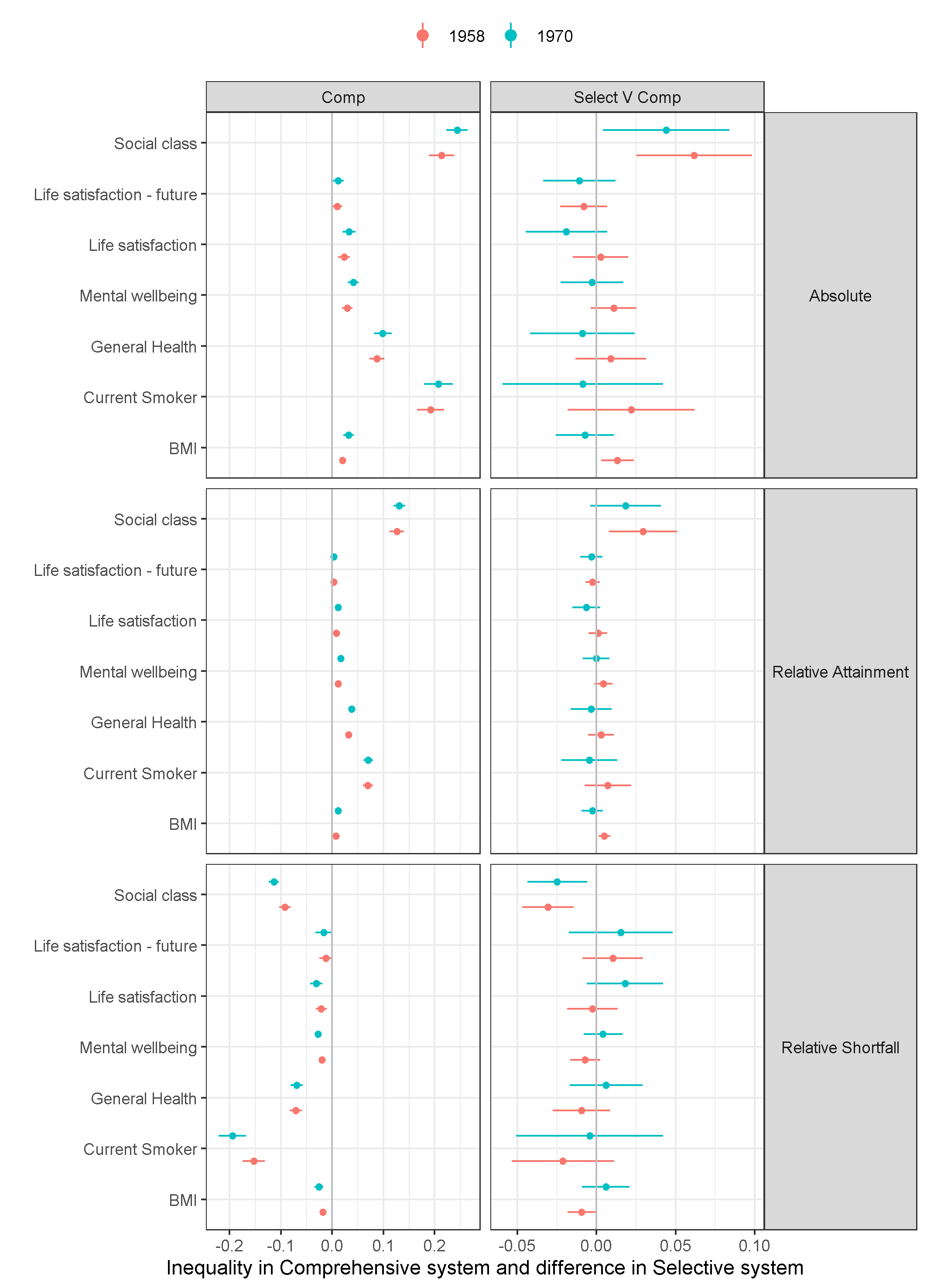


Figure S6_4 Education inequalities (age left education) in outcomes and social class by school system and cohort


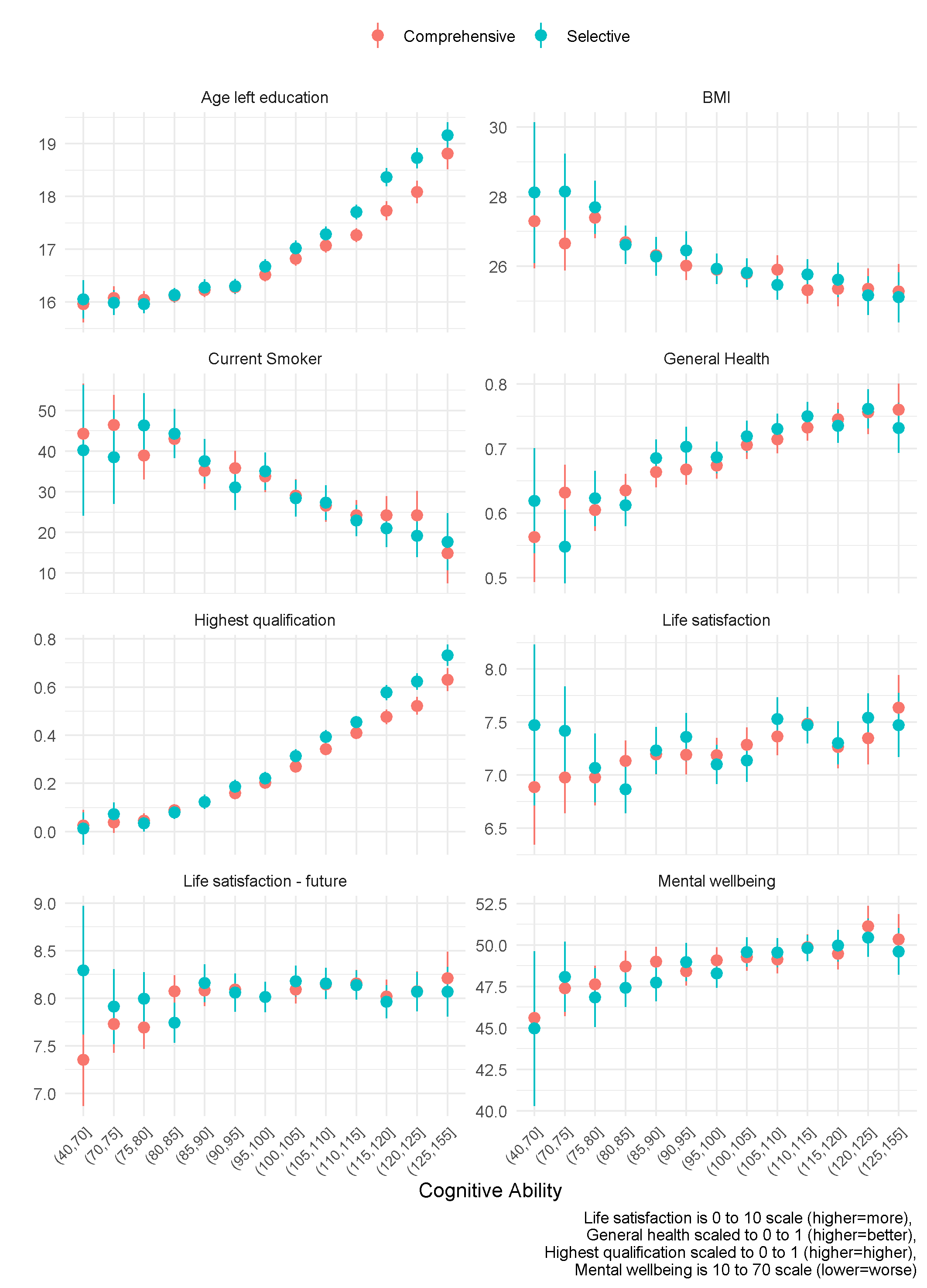


Figure S6_5 Mean of outcomes by cognitive ability and school system 1958 cohort

# Supplement 7 Analysis repeated with no imputed exposures


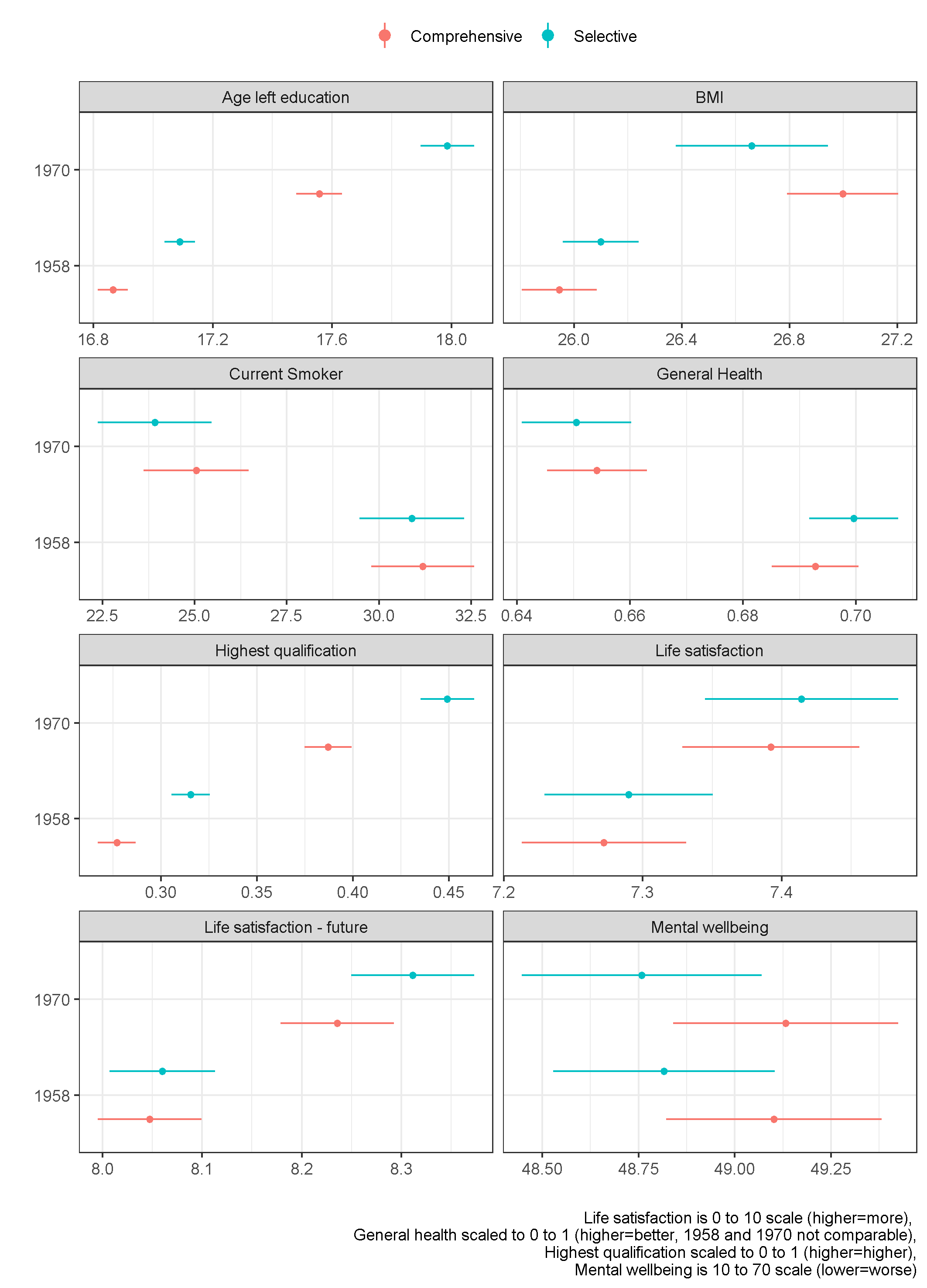


Figure S7_1 Mean outcomes by school system and cohort


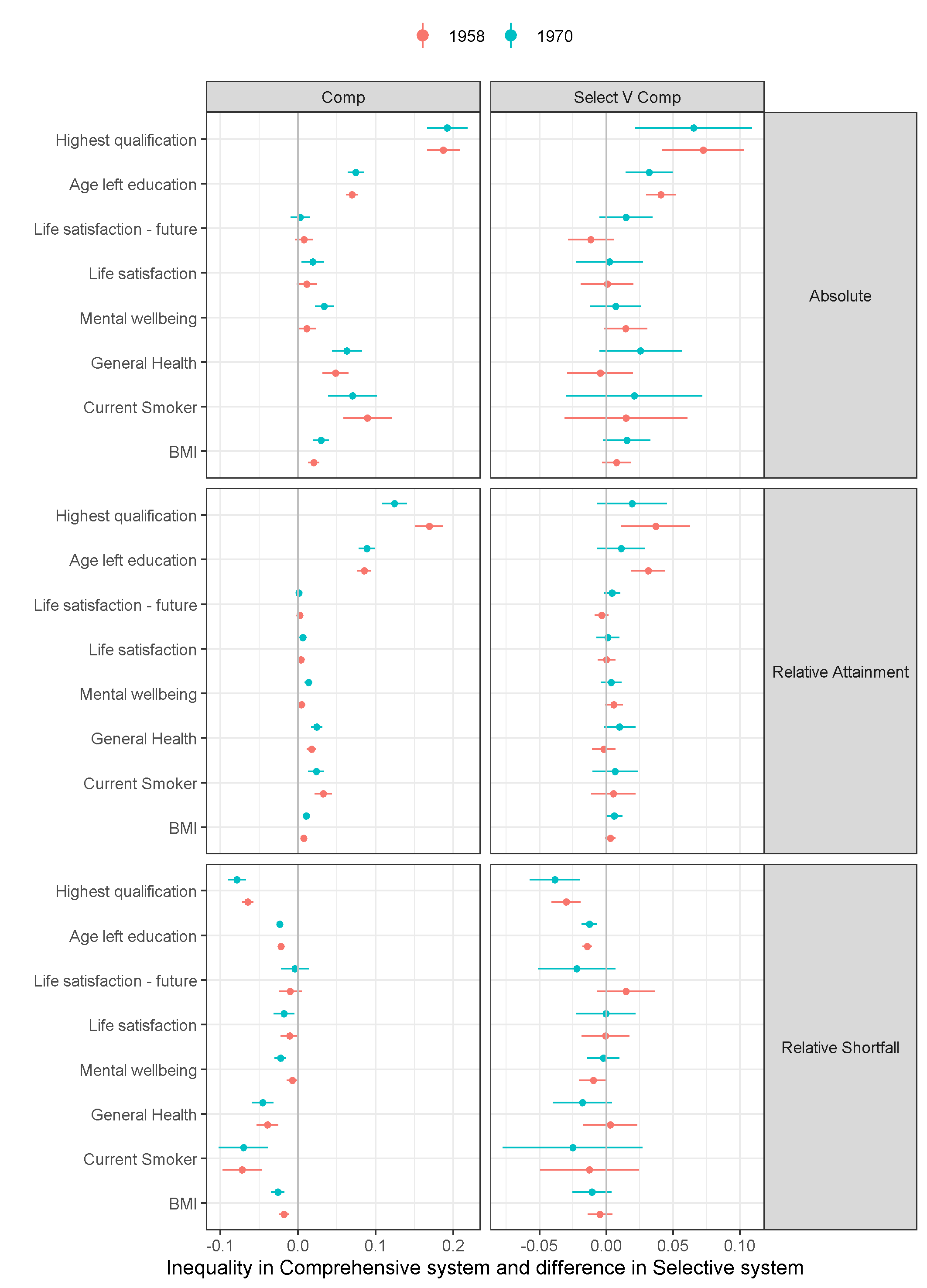


Figure S7_2)Origin class inequalities in outcomes by school system and cohort

# Supplement 8 Analysis repeated with 1958 school type being at 11 rather than 16


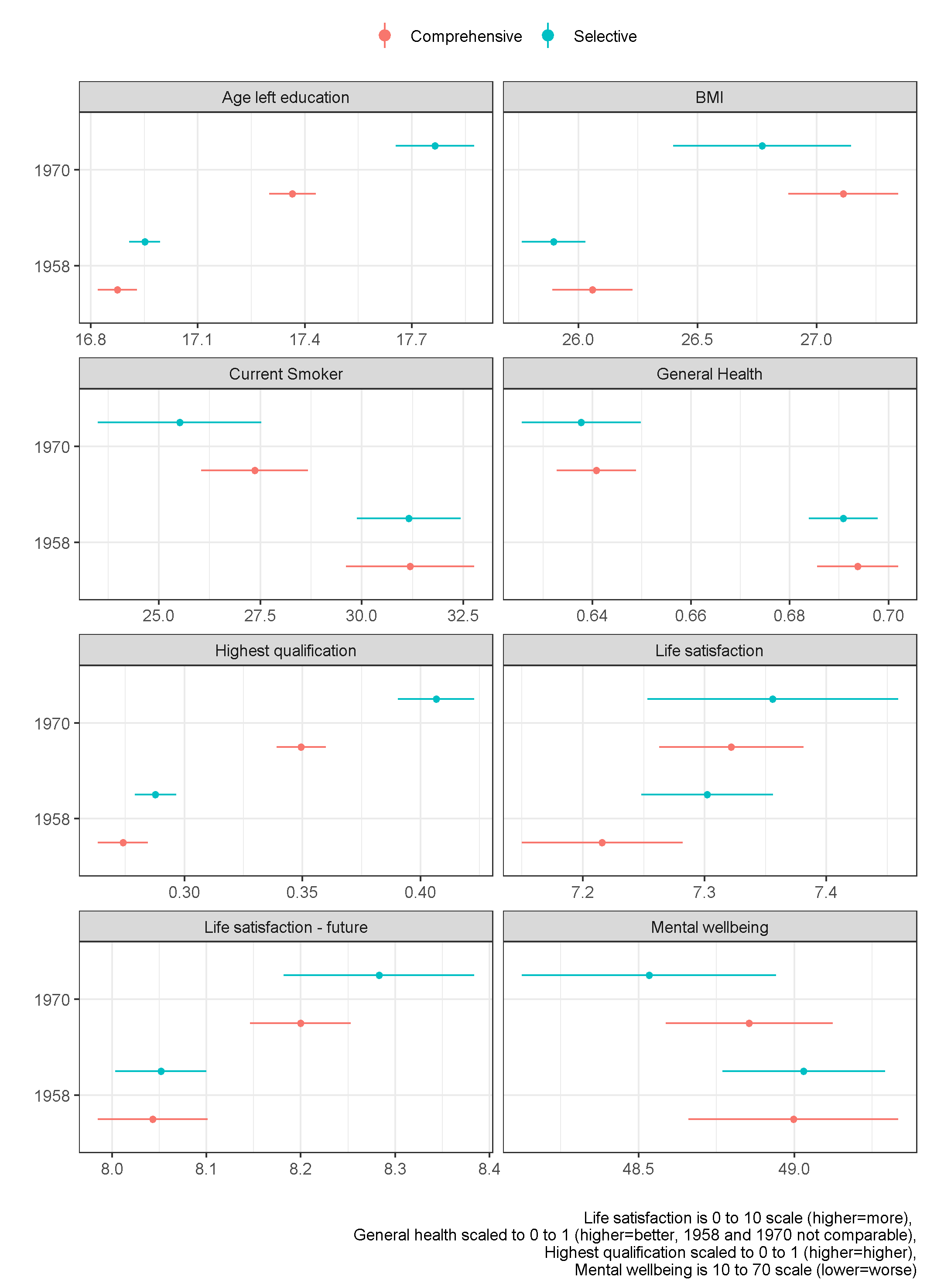


Figure S8_1 Mean outcomes by school system and cohort


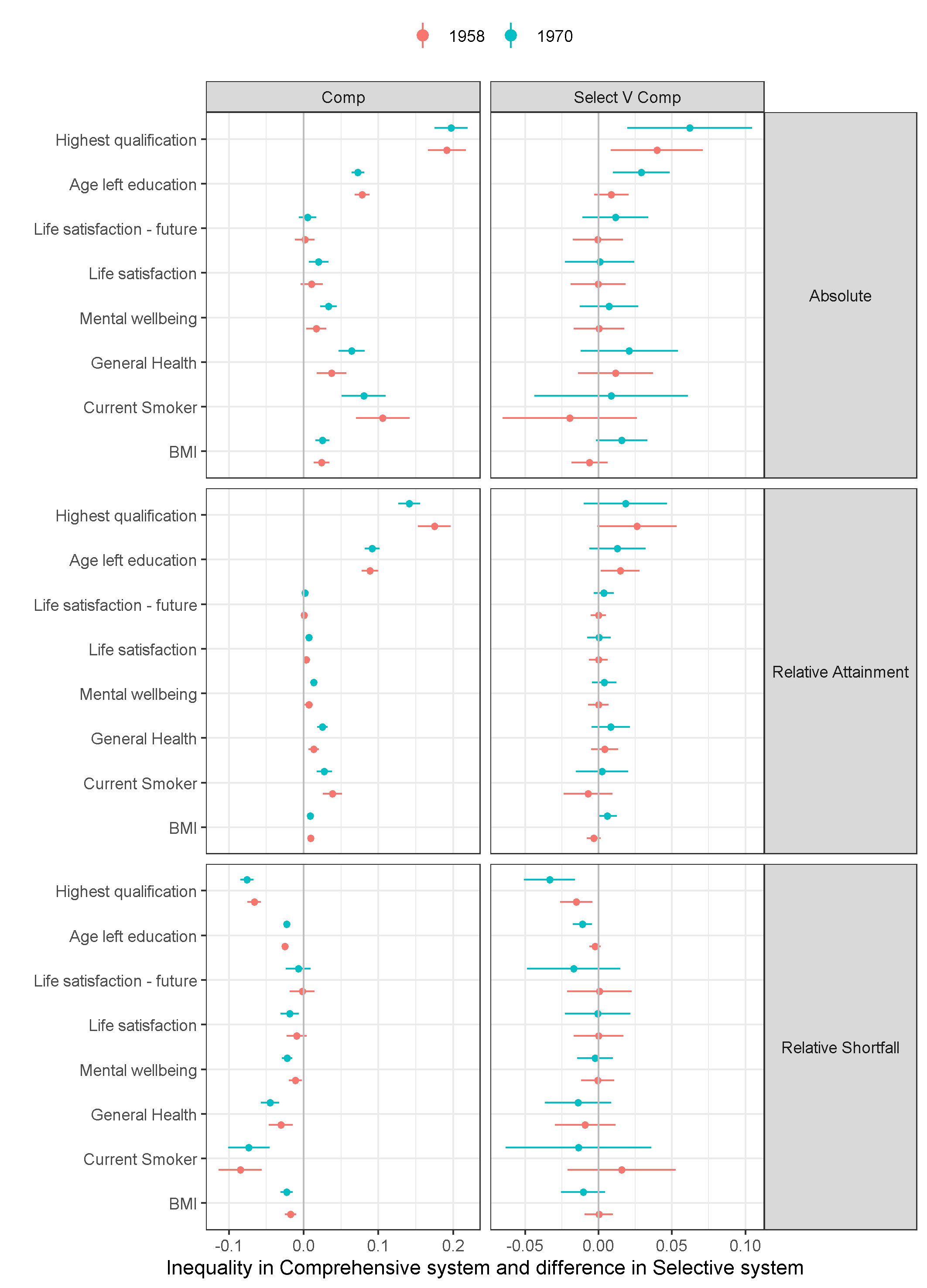


Figure S8_2 Origin class inequalities in outcomes by school system and cohort

# Supplement 9 R packages

## Wickham et al., (2019). Welcome to the tidyverse. Journal of Open Source Software, 4(43), 1686, https://doi.org/10.21105/joss.01686

## Yihui Xie (2020). bookdown: Authoring Books and Technical Documents with R Markdown. R package version 0.21.
##
## Yihui Xie (2016). bookdown: Authoring Books and Technical Documents with R Markdown. Chapman and Hall/CRC. ISBN 978-1138700109

## Yihui Xie (2020). knitr: A General-Purpose Package for Dynamic Report Generation in R. R package version 1.30.
##
## Yihui Xie (2015) Dynamic Documents with R and knitr. 2nd edition. Chapman and Hall/CRC. ISBN 978-1498716963
##
## Yihui Xie (2014) knitr: A Comprehensive Tool for Reproducible Research in R. In Victoria Stodden, Friedrich Leisch and Roger D. Peng, editors, Implementing Reproducible Computational Research. Chapman and Hall/CRC. ISBN 978-1466561595

## JJ Allaire and Yihui Xie and Jonathan McPherson and Javier Luraschi and Kevin Ushey and Aron Atkins and Hadley Wickham and Joe Cheng and Winston Chang and Richard Iannone (2020). rmarkdown: Dynamic Documents for R. R package version 2.6. URL https://rmarkdown.rstudio.com.
##
## Yihui Xie and J.J. Allaire and Garrett Grolemund (2018). R Markdown: The Definitive Guide. Chapman and Hall/CRC. ISBN 9781138359338. URL https://bookdown.org/yihui/rmarkdown.
##
## Yihui Xie and Christophe Dervieux and Emily Riederer (2020). R Markdown Cookbook. Chapman and Hall/CRC. ISBN 9780367563837. URL https://bookdown.org/yihui/rmarkdown-cookbook.

## David Robinson, Alex Hayes and Simon Couch (2020). broom: Convert Statistical Objects into Tidy Tibbles. R package version 0.7.3. https://CRAN.R-project.org/package=broom

## Stef van Buuren, Karin Groothuis-Oudshoorn (2011). mice: Multivariate Imputation by Chained Equations in R. Journal of Statistical Software, 45(3), 1-67. URL https://www.jstatsoft.org/v45/i03/.

## David Gohel (2020). flextable: Functions for Tabular Reporting. R package version 0.6.1. https://CRAN.R-project.org/package=flextable

## Noah Greifer (2020). WeightIt: Weighting for Covariate Balance in Observational Studies. R package version 0.10.2. https://CRAN.R-project.org/package=WeightIt
